# Direct and indirect costs associated with declining distance visual acuity: A nationwide longitudinal cost‐of‐illness study with 11‐year follow‐up

**DOI:** 10.1111/aos.70060

**Published:** 2026-01-27

**Authors:** Joonas Taipale, Petri Purola, Janika Nättinen, Saku Väätäinen, Seppo Koskinen, Hannu Uusitalo

**Affiliations:** ^1^ Department of Ophthalmology, Faculty of Medicine and Health Technology Tampere University Tampere Finland; ^2^ Tays Eye Center Tampere University Hospital Tampere Finland; ^3^ Finnish Register of Visual Impairment Finnish Federation of the Visually Impaired Helsinki Finland; ^4^ Finnish Medicines Agency FIMEA Tampere Finland; ^5^ ESiOR Oy Kuopio Finland; ^6^ Department of Public Health and Welfare Finnish Institute for Health and Welfare Helsinki Finland

**Keywords:** blindness, burden of disease, cost‐of‐illness, declining vision, health expenditure, longitudinal study, population‐based study, productivity losses, societal costs

## Abstract

**Purpose:**

To examine the direct and indirect costs associated with declining distance visual acuity (VA).

**Methods:**

A longitudinal approach was applied to a large, nationally representative sample with extensive data, including VA measured at two time points. The data of 3867 eligible participants aged 30 years or older at baseline were linked with national registers to estimate health care utilization from 1999 to 2013. A prevalence‐based bottom‐up approach was utilized to calculate the direct and indirect costs at the 2019 level. Data were adjusted for age, sex and incident comorbidities using generalized linear modelling (GLM). To estimate the cost‐of‐illness (COI), observed expenses among individuals whose VA declined during the follow‐up were compared to the counterfactual situation where VA had not declined, based on the regression analyses. Both individual and population‐level costs were reported.

**Results:**

The annual total direct health care costs per person were €976 for those with non‐declining VA and €1838 for those with declining VA (adjusted to match persons without decline in VA). The adjusted indirect costs were €14 579 and €22 631 for the working age subgroups, respectively. In counterfactual analysis, the annual COI associated with declining VA was estimated at €1166 from direct and €6411 from indirect cost sources. At the population level, the direct costs were €0.3 billion and the indirect costs, calculated for the working‐age population, were €0.4 billion annually.

**Conclusion:**

In total, 7.6% of the national direct health care costs were attributed to declining VA, highlighting the savings potential if the prevalence of declining VA could be reduced.

## 1 INTRODUCTION

Ageing populations, combined with continuously improving methods of diagnosing and treating various conditions, create an increasing need to understand the rising costs associated with diseases and functional limitations related to them. To effectively allocate limited health care resources and facilitate decision‐making, robust estimates assessing the burden of diseases on patients and society are required. Although data on the impact of various diseases on personal well‐being and health‐related quality of life (HRQoL), and also on societal and health care costs have been published, there is a gap in understanding how developing functional limitations, such as declining visual acuity (VA), affect them (Antonazzo et al., 2024, Frick et al., 2012, Jin & McCrone, 2015, Purola, Taipale, et al., 2023, Taipale et al., 2025). The lack of this type of data currently hinders a well‐informed decision‐making process.

Although the prognosis of many vision‐threatening conditions is improving and the functional limitation caused by them is occurring later than before (Purola et al., 2022; Purola, Kaarniranta, et al., 2023; Vaajanen et al., 2022), the predicted number of individuals with visual impairment is expected to increase, mostly due to ageing populations (Bourne et al., 2021; Wong et al., 2014). Another major challenge in the future will be globally increasing myopia prevalence, more prominent among high school students, females, and those residing in East Asian countries (Liang et al., 2025). Many burden‐of‐disease studies in the ophthalmological context have focused on the disease‐specific burden associated with various eye diseases instead of the functional limitation – declining VA (Purola, Kaarniranta, et al., 2023; Sonron et al., 2020; Tham et al., 2014; Wong et al., 2014; Xu et al., 2021; Zhang et al., 2023). Furthermore, prognoses among individuals can differ considerably even within a certain disease group. Therefore, examining also the burden associated with declining VA, in addition to the burden of individual diseases, can enhance our general understanding of the effects of declining VA regardless of the diagnosis.

We have recently published epidemiological data revealing the relationship between decreased VA and societal cost components (Purola, Taipale, et al., 2023; Taipale et al., 2025). Additional regional and global estimates on both direct and indirect costs associated with vision loss or blindness have been published (Chakravarthy et al., 2017; Gordois et al., 2012; Green et al., 2016; Köberlein et al., 2013; Marques et al., 2021; Pezzullo et al., 2018). Based on self‐reported visual limitations (‘Are you blind or do you have serious difficulty seeing, even when wearing glasses?’), Rein and colleagues reported an evaluation including multiple cost components, estimating the economic burden of vision loss and blindness in the United States (Rein et al., 2021). It should be noted that the vast majority of the increased health care costs associated with decreased VA in general or specific diseases, such as glaucoma, arise from sources other than ophthalmological care (Purola, Taipale, et al., 2023; Taipale et al., 2025). Along with decreased VA, an individual's productivity also decreases (Marques et al., 2021).

In addition to the awareness of the costs of existing functional impairment, the costs of developing functional impairment or new incident diseases are very valuable for evaluating health economic dynamics in societies and for fair and effective allocation of resources. However, there is a significant gap in our understanding. To our knowledge, only the intangible costs associated with longitudinally declining VA have previously been estimated in terms of reduced quality of life (Taipale et al., 2019).

In the present study, we utilize data from two nationally representative health examination surveys and national registers to examine how declining VA affects direct and indirect costs in a longitudinal setting. The utilized approach allows us to better account for other co‐occurring diseases, which may also contribute to health care usage during the observed period. Measured habitual distance VA is a more precise way to estimate actual VA than a questionnaire. This study provides urgently needed estimates of the direct and indirect costs associated with longitudinally declining VA during the 11‐year follow‐up period.

## 2 METHODS

### 2.1 Study population and survey design

The Finnish Institute for Health and Welfare conducted the Health 2000 study and its 11‐year follow‐up study, Health 2011, in Finland. These nationwide studies were designed to represent the adult population in Finland and included questionnaires and thorough health examinations. A weighting scheme was also designed to further increase the national population representativeness. More detailed descriptions of these studies have been published elsewhere (Heistaro, 2008; Lundqvist & Mäki‐Opas, 2016). STROBE guidelines were followed in reporting, where applicable (Von Elm et al., 2007).

Our study sample includes 3867 participants aged 30 or older at baseline, whose VA was measured at both time points. The direct costs were calculated for those participants with available data for each cost category and comorbidities. Register‐based analyses included 3803 participants, while the analyses for self‐reported cost categories included 1650 participants. The indirect costs were calculated for 1523 participants, aged 64 or less at baseline. Table 1 shows the number of participants with sufficient data for the analyses.

**TABLE 1:** Participants and available data according to distance visual acuity (VA) change during the follow‐up.

Number of participants with data available	Declining distance VA	Non‐declining distance VA^a^	Total
Total	298	3569	3867
The level of education, total	298	3558	3856
High level education	47	1287	1334
Middle level education	75	1259	1334
Low level education	176	1012	1188
Incident heart disease	291	3514	3805
Incident pulmonary disease	291	3514	3805
Incident vascular disease	291	3513	3804
Incident musculoskeletal condition	291	3514	3805
Incident hypertension	291	3514	3805
Incident diabetes	291	3514	3805
Incident psychiatric disorder	291	3513	3804
Incident Parkinson disease	291	3514	3805
Incident cancer	291	3514	3805
Pensionary status known (main primary function)	291	3512	3803
Self‐reported months of unemployment, lay‐off, and pension	298	3569	3867
Self‐reported costs in 2000	298	3558	3856
Self‐reported costs in 2011^b^	107	1551	1658
Register‐based costs	298	3569	3867

*Note*: A decline of at least 2 lines in the letter chart was classified as declining VA.

^a^ Includes participants whose distance VA improved during the follow‐up.

^b^ In total, 3805 participants out of 3867 (98.4%) answered to the first question of the questionnaire surveying outpatient health care utilization, but only 1658 completed it sufficiently comprehensively to enable cost analysis.

### 2.2 Visual acuity

A specially trained study nurse measured presenting distance and near VA binocularly in both time points, applying a modified logMAR letter chart with illumination of ≥350 lux. Distance VA was utilized in the analyses, as it has previously been found to have more impact on coping with daily activities and the quality of life of an individual (Taipale et al., 2019). Distance VA was classified according to prior studies: VA ≥1.0 as good vision, VA 0.63–0.8 as adequate vision, VA 0.32–0.5 as weak vision, and VA <0.3 as visual impairment or severe loss, including blindness (Taipale et al., 2019, 2025).

VA was considered declined or improved when a change of at least 2 lines, equaling 10 ETDRS characters, was observed in the VA chart between the baseline and follow‐up investigations. In previous studies with long follow‐up, this has been considered a clinically meaningful threshold (Taipale et al., 2019).

### 2.3 Health care utilization

The use of health services was estimated based on register data and questionnaires. The national HILMO and avoHILMO health care registers obtain the data from health care providers automatically. These registers consist of hospital outpatient visits and hospitalization periods, and the data are collected by the Finnish National Institute for Health and Welfare. To estimate the annual use of health services more precisely, we included all registered health care visits from 1.1.1999 to 31.12.2013 in our analyses and averaged these into annual costs. The register data were deterministically linked with participants' survey data via the national ID number.

Additionally, the use of ophthalmic care services was separately evaluated. Eye‐related hospitalizations were classified in the register data. Outpatient visits were considered eye‐related when the main diagnosis was ICD9‐coded with an eye‐related code (H0**–H5**).

The number of visits to a public primary care physician, occupational health care physician, occupational health care nurse, and private practitioner, as well as other visits to a physician, other visits to a nurse, and home visits by a nurse, were estimated based on the questionnaire. The participants reported their visits during the 12 months prior to assessment. Self‐reported data were available for 99.5% (3856 of 3876 participants) in 2000, and for 42.8% (1658 of 3876 participants) in 2011. The questionnaire also collected hospital outpatient visits data, but as these were captured more accurately with the health care registers, the register‐based data were analysed instead.

Emergency and non‐emergency outpatient visits to both primary and specialized health care have different average costs, and therefore, their shares of the total visits had to be estimated. As the shares were not distinguishable based on our data, the nationwide Sotkanet database was utilized to estimate the proportion of emergency visits in primary health care (Finnish Institute for Health and Welfare, 2024). For specialized health care, visits to Pirkanmaa Hospital District (PSHP) were estimated. This is considered to produce a reliable estimate on the secondary health care emergency visit proportion, as PSHP is one of the major hospital districts in Finland. The proportion of emergency visits was 37.2% in primary health care, 9.8% in specialized health care in general, and 8.4% for ophthalmologists. The weighted mean costs for outpatient visits were calculated based on these proportions.

Ninety (2.7%) out of the 3867 participants passed away during the follow‐up period (in or before 31.12.2013, but after the follow‐up visit in 2011). Therefore, the follow‐up time was calculated for each participant separately according to the actual follow‐up time to improve the estimates on average annual usage.

### 2.4 Health care costs

This study and the conducted analyses adhere to the CHEERS 2022 guidelines (see also the Data S1) (Husereau et al., 2022). We utilized a prevalence‐based bottom‐up approach to assess both direct and indirect costs. The expenses in cost‐of‐illness (COI) studies are usually stratified into three categories: direct, indirect, and intangible costs (Jo, 2014). Direct costs covered in our study include outpatient health care in primary and specialized care (general practitioners, specialists, and nurses), travel expenses to outpatient care, and hospitalizations. We estimated the indirect costs through early pension costs and productivity losses associated with early retirement, unemployment, or layoff. Intangible costs are observable but not easily measurable costs, such as psychological burden or decreased quality of life. Comprehensive estimation of these is not possible retrospectively and is not within the scope of the present study. We have estimated the decreased quality of life associated with declining VA in a longitudinal setting in our earlier publication (Taipale et al., 2019).

We applied the mean unit costs from Kelasto (Statistical database of the Social Insurance Institution of Finland) (The Social Insurance Institution of Finland, 2023) and a report published jointly by the Finnish Institute for Health and Welfare, the Social Insurance Institution of Finland, and Pharma Industry Finland (Kapiainen et al., 2014). The unit costs for outpatient visits to primary health care include customer fees. The travel expenses for outpatient care visits were estimated according to a previous publication (Väätäinen et al., 2019). The number of visits and hospitalization days were multiplied by the mean unit costs at the 2019 level to estimate the total cost for each category, and this total cost was divided by the follow‐up time of each participant to gain the average annual cost for every participant. The unit cost table has been previously published elsewhere and is reproduced here as Table 2 with the authors' permission (Taipale et al., 2025).

**TABLE 2:** Health care unit costs in 2011 or 2017, and 2019 levels.

Health care resource	Unit cost (€)		References
	In 2011	In 2019	
Primary health care physician visit (including patient fee, and collateral costs such as laboratory, imaging and other general costs)	105	129	Kapiainen et al. (2014)
During office hours	110	135	Kapiainen et al. (2014)
On emergency duty	96	118	Kapiainen et al. (2014)
Private practitioner (including administrative payment)	66	81	Kapiainen et al. (2014)
Occupational physician visit	77	95	Kapiainen et al. (2014)
Occupational nurse visit	28	34	Kapiainen et al. (2014)
Home care nurse visit	110	135	Kapiainen et al. (2014)
Outpatient nurse visit	48	59	Kapiainen et al. (2014)
Primary care hospital ward day	213	262	Kapiainen et al. (2014)
Secondary/tertiary somatic hospital ward day	737	905	Kapiainen et al. (2014)
Secondary/tertiary hospital ophthalmic ward day	873	1072	Kapiainen et al. (2014)
Psychiatric hospital ward day	408	501	Kapiainen et al. (2014)
Secondary/tertiary care outpatient visit to a physician	264	324	Kapiainen et al. (2014)
During office hours	263	323	Kapiainen et al. (2014), The Finnish Institute for Health and Welfare (2024)
On emergency duty	278	342	Kapiainen et al. (2014), The Finnish Institute for Health and Welfare (2024)
Secondary/tertiary care outpatient visit to an eye clinic	199	244	Kapiainen et al. (2014)
During office hours	201	247	Kapiainen et al. (2014), The Finnish Institute for Health and Welfare (2024)
On emergency duty	174	213	Kapiainen et al. (2014), The Finnish Institute for Health and Welfare (2024)
Annual pension	16 428	20 178	Finnish Centre for Pensions (2012)
Annual gross domestic product	36 746	45 133	Kuusisto (2019)
Travel cost per outpatient visit	**In 2017**	**In 2019**	
Southern Finland	18	19	Väätäinen et al. (2019)
Eastern Finland	30	32	
Northern Finland	41	44	
Central Finland	24	25	
Western Finland	22	23	
Other	25	26	

*Note*: Adapted from a previous publication with permission (Taipale et al., 2025). The costs were converted from 2011 or 2017 to the 2019 level based on national estimates on health expenditure and financing (Matveinen, 2021).

Kelasto data include the administrative costs for public but not for private health care. Therefore, we estimated the mean administrative costs in private health care by calculating the administrative costs for the ten most significant private health care service providers, weighting those according to their respective market shares in 2017, as more recent data were not available (Tevameri, 2018). After excluding those health care service providers concentrating in the oral or laboratory sector and accounting for the merging of some providers since 2017, three companies out of the original ten were left for the analyses. Calculated administrative cost was converted to the 2011 level according to the Finnish Cost‐of‐living index to make it compatible with Kelasto mean unit costs (Statistics Finland, 2024). After that, all the costs were converted to the 2019 level with the most recent estimates (Matveinen, 2021) to keep them compatible with our earlier research (Purola, Taipale, et al., 2023; Taipale et al., 2025).

### 2.5 Indirect costs

The following indirect costs were estimated: early retirement pensions, productivity losses due to early retirement, and productivity losses due to unemployment or layoff. Indirect costs were estimated based on questionnaire data. The participants were inquired about the number of months they had been unemployed or laid off during the last 5 years (the last 60 months), and, based on this, an average annual productivity loss was estimated. When participants' self‐reported principal activity was pensioner in the 2011 survey, the number of months being unemployed or laid off (if any) during the last 5 years was subtracted from sixty, and the remaining months were regarded as a maximum limit of retirement months. This approach was essential to avoid double‐counting the productivity loss by ensuring that a participant cannot be considered retired and unemployed at the same time. Next, the annual pension cost was calculated for each participant by multiplying the average yearly number of months of retirement by the average monthly pension (€1682 in 2019 level) in Finland (Table 2) (Finnish Centre for Pensions, 2012). Similarly, the number of months being unemployed or laid off was averaged at the annual level by multiplying them by the average gross domestic product (GDP) in 2011, inflated to the 2019 level (€3761) (Kuusisto, 2019).

The participants reported their year of retirement, according to which the lost working years were calculated. As those aged 65 years or older highly likely receive old‐age pension, early retirement was defined as lost working years between the ages from 30 to 64 years. For this reason, the indirect costs were calculated only for participants aged from 30 to 64 years at baseline. The retirement ages were supplemented based on participants' baseline distance VA for retired people without a reported year of retirement. The average reported retirement ages were 56.6, 57.5, 57.8, and 57.3 years for those with good, adequate, weak, or impaired VA in baseline, respectively. The productivity loss was conservatively estimated by applying GDP per capita, as was the case with the productivity losses due to unemployment or layoffs (Kuusisto, 2019). In order to report annual costs instead of lifetime expenses, the productivity loss associated with early retirement was divided by the mean duration of working career in Finland (32.6 years in 2011) (Järnefelt et al., 2013).

### 2.6 Data analysis and calculation of population‐level estimates

The data were analysed with SPSS Statistical Software version 29 (IBM Corp., Armonk, NY, USA), and the regression analyses were conducted with R software (v. 4.2.1, R Core Team, R Foundation for Statistical Computing, Austria) using packages twopartm 0.1.0 and margins 0.3.26 (Belotti et al., 2015; Leeper, 2021). Complex sampling design and the weighting scheme were accounted for by utilizing the Complex Samples module in SPSS and Survey package 3.37 in R (Koskinen et al., 2012; Lumley, 2004).

Generalized linear models (GLMs) were conducted to estimate the costs associated with each subset contributing to health care usage. Total direct and indirect costs were then added up based on these adjusted regression analyses. A large proportion of health care costs tends to be caused by a small proportion of the population with some individuals having no costs at all, meaning the distribution of costs is highly skewed and zero‐inflated. In such data, ordinary regression methods that assume normal distribution are not appropriate. Based on literature, maximum likelihood GLM using the Tweedie distribution with log link scale response was deemed appropriate for cost categories where the proportion of zero cost observations was ≤20%. When zero cost observations were more common, we applied two‐part gamma regression instead. (Kurz, 2017; Mihaylova et al., 2011) For two‐step regression models, different distribution families and parameters were tested, and the model with the best statistical fit, defined with the lowest Akaike's Information Criterion (AIC), was chosen. Bonferroni correction was applied to adjust for multiple comparisons. Ophthalmic inpatient care could not be evaluated separately with a GLM model due to a small proportion of participants with eye‐related hospitalization periods and sufficient data available on comorbidities, so these were analysed pooled to overall inpatient care. Additional subset analyses were performed to evaluate the robustness and sensitivity of our models for all indirect regressions and inpatient care, which was the most prominent direct cost category. These included subsamples of either sex, different age restrictions, and baseline VA levels.

Age, sex and the level of education were included in our GLM models as confounding factors with the incident diseases. The following incident diseases, based on self‐reported data, were included: heart disease, pulmonary disease, vascular disease, musculoskeletal condition, hypertension, diabetes, psychiatric disorder, and cancer. As baseline VA has an impact on how VA can change during the follow‐up period, it was included in the model as well. Further information about the selection and grouping of the variables is available in our previous publications (Purola, Taipale, et al., 2023; Taipale et al., 2019, 2025).

COI were estimated using a counterfactual approach. Counterfactual costs describe a hypothetical situation in which individuals with declining distance VA would, counter to the fact, not have had their VA decline during the follow‐up period, while everything else remaining the same (ceteris paribus). Counterfactual costs were estimated for each cost category separately using a simple parametric Monte‐Carlo simulation combining two sources of uncertainty: (1) the observed mean cost for the declining VA group and (2) adjusted declining vs. non‐declining effect from the regression models, adjusted for essential confounding factors and comorbidities.

First, using the observed mean and SE for the declining VA group, we generated many plausible values for its sample mean from a positive‐only Gamma distribution calibrated to match that mean and SE. To remove small simulation bias, we applied a one‐time calibration so that the simulated observed declining VA means matched the empirical observed mean and SE closely. Second, for each simulation run we drew a plausible value for the adjusted group effect (regression model coefficient for declining vs. non‐declining VA) from a normal distribution and removed the effect of declining VA to create counterfactual ‘no decline in VA’ mean (log link for positive‐cost models; logit link for any‐cost in two‐part models). For two‐part models, in each run we drew both the logit coefficient for any‐cost and the log‐Gamma coefficient for positive costs. The process was repeated 10 000 times; the SE for the counterfactual mean was the standard deviation of the simulated counterfactual means. For each category, the individual‐level COI was defined as the difference between the observed declining VA mean and the simulated counterfactual mean; uncertainty was obtained by repeating this difference within each simulation and summarizing its spread. National level COI was then estimated by multiplying the individual‐level estimated COI by the estimated number of those with declining VA in the Finnish population.

The population estimates are based on the observed changes in VA status, population weights included in the data, and national population demographics on 31.12.2011, provided by the Official Statistics of Finland (Statistics Finland, 2023). In more detail, the weighted proportions of participants with declining VA in different age groups (30–44, 45–54, 55–64, 65–74, and 75 or older) were calculated according to our data and were then extrapolated to the population level for each of these age groups. The number of those with declining VA in the Finnish population in total was the sum of these subsets.

### 2.7 Informed consent and ethical approval

All participants signed two agreements beforehand on both time points, one for questionnaires and the other for the health examination. Ethical approval process details have been described in previous publications (Heistaro, 2008; Lundqvist & Mäki‐Opas, 2016). This study adhered to the tenets of the 1964 Helsinki declaration and its later amendments.

## 3 RESULTS

A total of 298 participants, 7.7% of those with completed VA examination (*n* = 3867), lost at least two lines in the VA chart, equaling ten ETDRS letters, during the 11‐year follow‐up period (considered as declining distance VA). In total, 51.7% (154/298) of them lost two lines, 28.5% (85/298) lost three lines, and 19.8% (59/298) lost at least four lines during the follow‐up period. Among these, approximately 95% (224/235) of the respondents wore glasses or contact lenses regularly according to the baseline questionnaire, while during the follow‐up visit, 91% (98/108) of the respondents reported wearing glasses.

The mean ages of the participants in 2011 were 72.4 years, 59.6 years, and 60.6 years for those with declining distance VA, non‐declining distance VA, and in total, respectively. The respective proportions of females were 57.0%, 55.2%, and 55.3% for these groups.

The observed mean hospital stay duration was 6.6 (standard error [SE] 0.92) days for those with declining VA, and 2.8 (SE 0.10) days for those without VA decline. Incidence of traumatic injury was 0.03 in both groups, and the incidence of bone fractures was 0.04 and 0.03 for declining and non‐declining groups, respectively.

### 3.1 Incremental costs of declining distance VA

Those with declining distance VA bore 88% higher total direct health care costs annually (€1838 vs €976) compared to those with non‐declining VA (Table 3). Interestingly, eye‐related outpatient care only accounted for 1.2% of the total direct health care costs in the non‐declining VA group, and for 1.1% in the declining VA group. Regardless of whether distance VA declined or not during the follow‐up, inpatient care accounted for the largest share of the direct costs (Table 3). In total, 63% (€1166) and 46% (€447) of the direct costs were from inpatient care for the declining and non‐declining groups, respectively.

**TABLE 3:** The average annual costs (€) per person during the follow‐up according to the change in visual acuity (VA) during the follow‐up.

Average annual costs per person, €	Non‐declining distance VA^a^, observed	Declining distance VA^b^, observed	Observed difference between groups	Adjusted difference, AME (see Tables S1–S9)	Implied adjusted cost with declining distance VA^b^
Total primary, secondary, and psychiatric inpatient care	447.39 (25.54)	1550.25 (175.73)	1102.86 (177.58)*	718.94 (152.24)*	1166.33 (154.37)*
Total secondary outpatient care	277.74 (8.24)	493.35 (57.91)	215.61 (58.49)*	140.21 (44.94)*	417.95 (45.69)*
Eye‐related secondary outpatient care	11.42 (0.78)	35.38 (4.89)	23.96 (4.95)*	8.69 (3.51)*	20.11 (3.60)*
Physician: Primary, occupational, and private care	142.05 (5.73)	67.32 (21.49)	−74.73 (22.24)*	−28.73 (40.29)	113.32 (40.70)*
Nurse: Primary, occupational, and private care	85.82 (13.96)	418.56 (162.89)	332.74 (163.49)*	21.77 (30.12)	107.59 (33.20)*
Travel expenses	22.65 (0.68)	39.56 (5.08)	16.91 (5.13)*	10.17 (3.66)*	32.82 (3.72)*
**Total direct health care costs for all participants**	**975.65 (36.05)**	**2569.04 (290.12)**	**1593.39 (292.40)** *	**862.36 (188.49)** *	**1838.01 (192.13)** *
Early retirement pensions	4040.38 (126.46)	5647.06 (402.10)	1606.68 (421.52)*	2355.72 (530.42)*	6396.10 (545.29)*
Productivity losses due to early retirement	9037.49 (282.86)	12631.30 (899.41)	3593.81 (942.84)*	5269.16 (1186.44)*	14306.65 (1219.69)*
Productivity losses due to unemployment or layoff	1500.80 (108.61)	2050.59 (464.66)	549.79 (477.18)	427.49 (730.48)	1928.29 (738.51)*
**Total indirect costs for participants under 65 years at the baseline**	**14578.67 (430.14)**	**20328.95 (1409.66)**	**5750.28 (1473.91)** *	**8052.37 (1908.28)** *	**22631.04 (1956.32)** *

*Note*: ‘Non‐declining distance VA, observed’ and ‘Declining distance VA, observed’ columns presents the observed crude mean values for those with non‐declining distance VA (including those with improved VA) and declining VA, correspondingly with their standard errors (SE) in parentheses, while ‘Adjusted difference’ (AME) column shows the adjusted regression estimates, calculated with the average marginal effects (AMEs). Each row, except the bolded ones, shows the results of a single GLM regression analysis adjusted for the essential confounding factors and comorbidities specified in Table 1. Regression analyses are presented in more detail in Tables S1–S9. The comorbidity prevalences and other covariates are standardized at the average level of those with non‐declining distance VA (including those with improved VA), so the only difference between the observed costs of non‐declining VA group and adjusted declining VA group is the VA change during the follow‐up period. For each cost category, the implied adjusted costs for the declining distance VA group were estimated as the sum of the average adjusted AME contrast and the average observed cost for the non‐declining distance VA group. The implied SE of the mean costs were estimated as the root of sums of squared AME SE and squared non‐declining VA group SE, assuming independence. For the total sums of direct and indirect costs, the SEs were estimated using the Pearson correlation matrix among the cost categories included in the sum. The costs are presented at 2019 levels. The direct costs were calculated for all participants, while the indirect costs were only calculated for those under 65 years of age at baseline.

^a^ Includes participants whose distance VA improved over time.

^b^ Participants who lost at least two lines in the VA chart, equaling ten ETDRS letters during the 11‐year follow‐up period.

* Statistically significant with *p* < 0.05.

Also, the indirect costs increased with declining distance VA. While the total indirect costs were €22 631 (SE 1956) per year on average among those with declining distance VA and less than 65 years of age at the baseline, the corresponding costs were €14 579 (SE 430) among those with non‐declining distance VA and less than 65 years of age at the baseline.

Utilized methodological approach allows us to compare the marginal effects of declining distance VA to other health conditions included in the model. The average additional annual direct costs associated with pulmonary diseases were the highest of the health conditions included in the model, with an annual average of €960 (SE 172), followed by the costs associated with declining distance VA (€862 annually, SE 188) (Table 4). However, the indirect costs were significantly higher for individuals whose distance VA declined during the follow‐up period compared to the other variables included.

**TABLE 4:** The average estimated annual direct and indirect costs (€) per person during the follow‐up for those with new incident conditions at some point during the follow‐up.

Condition occurred during follow‐up	Annual cost‐of‐illness, € (standard error, SE)	
	Direct costs	Indirect costs
Incident heart disease	791.45 (121.57)	436.10 (1058.50)
Incident pulmonary disease	960.31 (172.15)	1950.50 (1448.42)
Incident vascular disease	727.59 (178.09)	1855.42 (1749.56)
Incident musculoskeletal condition	256.84 (62.15)	−1144.99 (808.25)
Incident hypertension	181.95 (79.06)	399.37 (1066.28)
Incident diabetes	852.13 (192.83)	3856.73 (1498.31)
Incident psychiatric disorder	579.23 (172.97)	2528.73 (2165.43)
Incident cancer	751.37 (185.83)	−702.69 (1514.24)
Declining distance VA	862.36 (188.49)	8052.37 (1908.28)

*Note*: Each row shows the aggregate direct and indirect costs based on the GLM regression analyses adjusted for the essential confounding factors and comorbidities specified in Table 1. The comorbidity prevalences for others than the covariate under consideration are adjusted to the average level observed in those with non‐declining VA. The SEs for the adjusted total AMEs for direct and indirect costs were estimated using the Pearson correlation matrix among the cost categories included in the sum. The costs are presented at 2019 levels.

The average marginal effects (AMEs) for each cost category and explanatory variable are reported in more detail in Tables S1–S9. As an example, of the included explanatory variables, incident pulmonary disease had the second‐highest effect on the inpatient care costs (€610, SE 130) after declining distance VA (€719, SE 152), whereas incident heart disease (€412, SE 82), vascular disease (€486, SE 140), and diabetes (€249, SE 102) had smaller magnitudes of effect. Those participants with better baseline VA (€ −101, SE 28) or a high level of education (€ −144, SE 51) had lower inpatient care costs instead (Tables S1).

### 3.2 Potential savings of declining distance VA

Another method utilized to evaluate the COI was to calculate the counterfactual costs for the participants whose distance VA declined during the follow‐up. Although this calculation does not facilitate comparison to other explanatory variables, the advantage of this approach is that it provides a monetary estimate of potential savings if distance VA had remained the same or improved instead of the observed decline.

At the individual level, the average additional annual direct costs of declining distance VA were estimated at €1166 (SE 230), and about 80% (€932, SE 136) of these were due to inpatient care (Table 5). For individuals aged less than 65 years at the baseline, the average additional indirect costs were estimated at €6411 (SE 1272), with early retirement being the major contributor for them.

**TABLE 5:** Observed, counterfactual and estimated annual additional costs (cost‐of‐illness) (€).

Annual costs	Observed costs of declining VA^a^, per person, as observed in the data (€)	Simulated counterfactual costs (no decline in VA), per person, (€)	Estimated cost‐of‐illness per person (€)	Estimated cost‐of‐illness at the national level (million €)	Cost‐of‐illness as a proportion of estimated total national level costs (%)
Total primary, secondary, and psychiatric inpatient care	1550.25 (175.73)	618.22 (112.04)	932.01 (135.77)	231.34 (33.70)	12.6
Total secondary outpatient care	493.35 (57.91)	337.07 (52.79)	155.87 (39.77)	38.69 (9.87)	3.8
Eye‐related secondary outpatient care	35.38 (4.89)	20.08 (4.56)	15.29 (4.14)	3.80 (1.03)	8.3
Physician: Primary, occupational, and private care	67.32 (21.49)	100.10 (47.03)	−32.83 (37.05)	−8.15 (9.20)	−1.7
Nurse: Primary, occupational and private care	418.56 (162.89)	319.38 (177.84)	99.08 (135.66)	24.59 (33.67)	6.4
Travel expenses	39.56 (5.08)	28.07 (4.69)	11.46 (3.37)	2.84 (0.84)	3.4
**Total direct health care costs for all participants**	**2569.04 (290.12)**	**1402.83 (255.60)**	**1165.58 (230.23)**	**289.32 (57.15)**	**7.6**
Early retirement pensions	5647.06 (402.10)	3757.25 (468.20)	1885.99 (345.22)	130.64 (23.91)	1.3
Productivity losses due to early retirement	12631.30 (899.41)	8405.90 (881.64)	4219.36 (717.31)	292.27 (49.69)	1.3
Productivity losses due to unemployment or layoff	2050.59 (464.66)	1743.40 (775.65)	305.48 (634.99)	21.16 (43.99)	0.6
**Total indirect costs for participants under 65 years at the baseline**	**20328.95 (1409.66)**	**13906.55 (1599.23)**	**6410.82 (1272.18)**	**444.07 (88.12)**	**1.2**

*Note*: Data are shown as mean (standard error, SE), at 2019 euros, unless otherwise indicated. The cost‐of‐illness quantifies the savings in a counterfactual scenario where visual acuity (VA) did not decline during follow‐up. Counterfactual costs were estimated for each cost category separately using simple parametric Monte‐Carlo simulation combining two sources of uncertainty: (1) the observed mean cost for the Declining VA group and (2) adjusted declining vs. non‐declining effect from the regression models (adjusted for essential confounding factors and comorbidities; reported in Tables S1–S9). First, using the observed mean and SE for the Declining VA group, we generated many plausible values for its sample mean from a positive‐only Gamma distribution calibrated to match that mean and SE. To remove small simulation bias, we applied a one‐time calibration so that the simulated observed Declining VA means matched the empirical observed mean and SE closely. Second, for each run we drew a plausible value for the adjusted group effect (model coefficient for declining vs. non‐declining VA) from a normal distribution and removed the effect of declining VA to create counterfactual “no decline” mean (log link for positive‐cost models; logit link for any‐cost in two‐part models). For two‐part models, in each run we drew both the logit coefficient for any‐cost and the log‐Gamma coefficient for positive costs. The process was repeated 10 000 times; the SE for the counterfactual mean was the standard deviation of the simulated counterfactual means. For each category, the cost‐of‐illness was defined as the difference between the observed Declining VA mean and the simulated counterfactual mean; uncertainty was obtained by repeating this difference within each simulation and summarizing its spread. National level cost‐of‐illness was calculated at the population level in 2011 according to Statistics Finland and the stratified cluster design weights by the Finnish Institute for Health and Welfare. The estimated number of citizens with declining distance VA was 248 217, and 69 269 of them were aged under 65 at the baseline.

^a^ Participants who lost at least two lines in the VA chart, equaling ten ETDRS letters, during the 11‐year follow‐up period.

The estimated number of citizens with declining distance VA was 248 217, and 69 269 (27.9%) of them were under 65 years of age, based on the national population demographics in 2011 and the stratified cluster design weights. The national level annual costs of declining distance VA were calculated based on these numbers and were estimated to be €0.7 billion, of which €0.3 billion (39.4%) as direct health care costs and €0.4 billion (60.6%) as indirect costs (Table 5).

The proportions of the total national costs attributed to declining distance VA were estimated by dividing the total national observed costs by the estimated COI. The additional costs attributed to declining distance VA cover a total of 12.6% (€231 million) of the national inpatient care costs (€1.8 billion). Furthermore, only 8.3% of eye‐related outpatient care costs were attributed to declining distance VA during the follow‐up period, which is only slightly more than the average for all direct health care costs (7.6%). The national level cost estimates for each category are reported in Table S10.

## 4 DISCUSSION

This study reports the costs associated with declining distance VA in a longitudinal setting, adjusted for socioeconomic factors and other incident diseases over the 11‐year follow‐up period. Our results indicate a correlation between declining distance VA and increased direct and indirect costs, even after controlling for multiple comorbidities. The employed methodology further facilitates a comparison of the costs linked to declining distance VA with those associated with other health conditions. While the direct costs related to declining distance VA ranked as the second highest among the variables examined, the indirect costs were the highest, as were the total costs. This comparison with other health conditions underscores the importance of distance VA in relation to overall expenses.

The included cost components encompass a wide array of essential expenses, thereby making further research and comparisons to findings in subsequent studies more attainable. The findings can assist in decision‐making and policy formulation by facilitating and informing, for instance, future cost‐effectiveness analyses concentrating on various procedures maintaining VA, and to advance the initiative to sustain VA at the best possible level, both at the individual and population levels.

In our regression models, male sex was associated with lower costs for all direct cost categories, although statistical significance was found only for secondary outpatient care, primary, occupational and private care physician and nurse visits. The level of education also showed a reducing trend in the costs, although this finding was not statistically significant for most of the categories. These, however, suggest lower overall annual health service utilization among males and those with higher education, albeit most likely, for different reasons. Better baseline distance VA was also associated with lower costs in all direct cost categories, excluding primary, occupational, and private care physician visit costs. See also our previous publication with a cross‐sectional focus on the costs associated with distance VA (Taipale et al., 2025). The results are consistent with previous findings, according to which male sex and a higher socioeconomic status are associated with lower costs, although previous research only considers visual impairment, specific eye diseases, or decreased VA without a longitudinal approach (Rein et al., 2021; Sonron, Tripathi & Hariharan 2020; Taipale et al., 2025; Xu et al., 2021).

At the individual level, the direct costs would have been €1403 per year instead of the observed €2569, if VA had not declined during the follow‐up. Thus, without declining distance VA, almost one‐half of the direct costs would have hypothetically been avoided. Our sensitivity analysis with hospital outpatient costs shows that for those with baseline VA ≤0.5, additional VA decline was not associated with an increase in hospital outpatient costs. This indicates that, especially for those with VA >0.5, maintaining optimal VA is beneficial concerning also the economic aspects.

While those with declining distance VA are somewhat older and have more incident comorbidities, these factors are already adjusted for in our estimate. In our data, self‐reported health care utilization (primary, occupational, and private health care physician and nurse visits) and their costs associated with declining VA were statistically insignificant after adjusting for comorbidities. However, participants with incomplete self‐reported health care utilization data were older (66.46 vs. 52.25 years at baseline) with more comorbidities compared to those with complete data on these variables, which might have reflected in our estimates presented. The prevalence of declining VA was also slightly higher among those with incomplete data compared to those with complete data, suggesting that incomplete data might have been associated with declining VA in addition to potentially greater impact of older age accompanied by additional comorbidities. We recommend further analysis to estimate whether longitudinally declining VA is associated with changes in these costs.

Previous research has shown that those with vision‐affecting diseases are more prone to falling, and the risk for fractures is increased (Tsang et al., 2024). In addition, the risk of increased hospitalization, particularly among older patients, is higher for those with lower VA (Morse et al., 1999, 2019). Our data showed no statistically significant differences in the total number of fractures or traumatic injuries between the groups with and without VA decline. However, those with declining VA more commonly had pelvic or femoral fractures, whereas those with non‐declining VA had more fractures in the upper and lower extremities. In our data, the average duration of a hospital stay was also considerably longer among those with declining VA compared to those without VA decline, which may result at least in part from these differences with traumatic injuries. Also, for those with declining VA, discharging from a hospital after a treatment period might be delayed, as the means for coping at home are weakened. Considering that inpatient care costs account for three‐quarters of the increased direct health costs, these factors together likely explain most of the excess costs. Increased hospitalizations also often reflect in increased outpatient health care follow‐up visits, both in specialized and primary health care.

Another interesting finding is that concerning the national level costs, only 8.3% of the eye‐related secondary outpatient care costs were attributed to declining distance VA during the follow‐up, while 92% were attributed to other factors. We speculated multiple explanatory factors contributing to this. First, only 7.1% (248 217/3 506 200) of those with at least 30 years of age were estimated to have declining VA during the follow‐up, which limits the number of people who were able to influence the costs associated with declining VA. Second, a wide range of diseases have ophthalmic manifestations, all of which do not necessarily affect VA, specifically when treated, but which might cause ocular problems and increase the need for ophthalmic care. Third, as binocular VA was measured, the costs are not associated with declining distance VA if the better‐seeing eye remains intact.

Most of the individual‐level costs due to declining distance VA originate from indirect cost sources. The average indirect costs for working‐aged participants with declining VA were over €22 631 annually, which was approximately half of the annual GDP per capita (€45 133). Productivity losses accounted for 71.7% of these, whereas early pensions accounted for the rest 28.3%. According to the chosen valuation methodology, individuals who were completely out of the workforce had productivity losses equaling GDP per capita, but in addition, early pension costs led to an even higher financial burden. Previous cross‐sectional research reported productivity loss associated with visual impairment or blindness to be 0.16% of the GDP in Western Europe and 0.32% globally (Marques et al., 2021). Although longitudinal comparators are unfortunately not yet available, the result of our study is somewhat conservative compared with their estimates as the total productivity losses of €313 million accounted for only 0.13% of the national GDP in 2019 (€239.9 billion, according to Statistics Finland). This may also be explained by the fact that no information regarding presenteeism and absenteeism was collected in our register data.

Around €6411 annual indirect costs could be spared for each person aged less than 65 years of age at the baseline with declining distance VA if distance VA did not decline. Those with declining VA are more likely to face challenges in working life, which leads to both reduced productivity while working and early retirement.

The estimated national population‐level cost of declining distance VA was €730 million annually, 39.4% of which was from direct health care costs and 60.6% was from indirect cost sources. The results are somewhat different compared to the findings in our previous study, in which the indirect costs associated with lower VA status were higher at the individual level, but in population‐level analysis, the direct costs constituted a larger share of the total costs (Taipale et al., 2025). In this study, however, longitudinally declining VA was associated with higher indirect costs also at the population level, after adjusting for multiple comorbidities. Declining VA might be a more important indicator of impaired occupational ability compared to decreased but stable VA; therefore also leading to more significant productivity losses at the population level. In a study from the United States, the direct costs accounted for 73.5% of the total costs associated with self‐reported blindness or serious seeing difficulties, even with glasses (Rein et al., 2021). As another study from Ireland reports the health care costs due to blindness at approximately to be from seven to eight percent of the productivity loss burden for those with blindness, the social, economic and geological context each have important impact on the obtained results (Green et al., 2016). In addition, the choice of cost categories included has an impact on the total cost calculation and their relative proportions as well.

According to previous research, about 40% of those with visual impairment receive informal care, and lower VA in general raises this probability (Marques et al., 2018). Including informal care could be beneficial in capturing the total burden more accurately, but this information was not included in our data. On the other hand, absenteeism due to sick leave or presenteeism was not integrated with our data, which reduces the amount of estimated indirect costs.

Some additional limitations need to be addressed as well. First, earlier research suggests that blindness causes substantial direct costs during the first year after its onset, but after that, the costs do not differ from those of non‐blind people (Frick et al., 2008). As our data comprises measured VA at two time points, but the costs accrue over the entire follow‐up time, we could not differentiate whether this is the case in our data as well.

Second, as the standard retirement age might have been somewhat lower than 65 years for some of the participants, our analyses might overestimate the costs of early retirement. However, assuming that participants with lower standard retirement age are distributed at random, the comparisons between the groups (declining or non‐declining VA) or between the observed and counterfactual costs are not affected.

Third, the data in the present study are from 1999 to 2013, and one might raise a question of how adequately these data correspond to the present situation. While opening and reusing pre‐existing data is now a growing trend in science, analysing existing data with multiple sources integrated in it can be considered ethical in comparison to collecting a new dataset. The unit cost data and population estimate from 2011 were applied in the estimates to improve compatibility with previous research. Also, the methodology applied in our study sharpens the estimates and is applicable as such to other datasets to produce precise estimates in different populations in the future.

Fourth, as habitual distance VA was measured instead of best‐corrected VA, for some participants, the observed decline could be due to inappropriate or forgotten glasses on the testing day. However, less than five participants (1.3%), whose VA declined during the follow‐up and who wore glasses at baseline, did not regularly wear them at the follow‐up visit. Furthermore, some of them had late‐stage macular degeneration, in which case wearing glasses generally does not affect VA. The use of inappropriate refractive correction, however, was not retrievable in our data, so some uncertainty might remain.

However, measuring VA with habitual correction instead of best‐corrected VA describes more accurately the everyday performance. If the participants do not wear appropriate glasses or their VA is otherwise impaired, they have an increased risk for injuries, such as falls, potentially leading to hospitalization, thereby contributing to increased costs (Tsang et al., 2024). Utilizing best‐corrected VA, in turn, fails to capture this increased risk.

The presented results arise from a retrospective setting, allowing only identification of correlations rather than causal relationships. Reported results should be supplemented by other studies with a prospective setting to evaluate the possibility of a causal relationship between declining VA and associated costs. Furthermore, although the analyses accounted for multiple comorbidities, the possibility of residual confounding cannot be ruled out with complete certainty. Also, not all cost categories showed statistically significant association with declining VA (self‐reported physician and nurse costs, and productivity losses due to unemployment or layoffs). However, these cost sources only make up a very small proportion of the total costs, and including them helps identify how declining VA constitutes its overall economic burden.

There are also other major advantages in our study. The data represent the adult population in Finland, and the applied weighting scheme further minimizes the nonresponse bias. Compared to patient cohort studies, our data help us to elude the potential bias of non‐healthy control participants. The study design and the unit costs calculated and reported earlier allow us to apply a prevalence‐based bottom‐up approach (Heistaro, 2008; Kapiainen et al., 2014; Lundqvist & Mäki‐Opas, 2016). As a prevalence‐based approach is the most common method in COI studies, utilizing it will facilitate comparison and possibly aggregating the reported results from multiple studies in the future (Jo, 2014). We also integrated the data from nationwide register databases to more accurately collect the costs associated with declining VA.

The authors are not aware of previously published studies estimating the costs of declining VA in a longitudinal setting with measured VA. Most of the previously conducted studies focus on vision impairment and blindness, as in a study from Germany, according to which decreasing vision was associated with higher costs (Chuvarayan et al., 2020). However, their period under review was just 6 months of retrospective costs, and only participants with visual impairment or blindness were included, limiting the generalizability. In addition, Pablo and colleagues have presented some projective estimates of future costs associated with vision loss and blindness in Spain (Pablo et al., 2024). However, projective estimates often fail to achieve the precision of collected data, as simulated projections are always more susceptible to factors that cannot be accounted for beforehand.

## 5 CONCLUSION

Our study highlighted the association between declining distance VA and both direct and indirect costs. The cost analyses with two complementary methodologies provide a good opportunity for comparison with future COI research on declining distance VA while also allowing for comparison to other variables in the model. The increased costs originate widely from various sources of the health care system due to the disruptive impact of declining VA on everyday life. One twelfth of the total national direct health care costs was attributed to declining VA, highlighting the potential national savings if the decline of distance VA could be prevented.

## Supporting information


Tables S1–S10.

Data S1.

## References

[aos70060-bib-0001] Antonazzo, I.C. , Gribaudo, G. , La Vecchia, A. , Ferrara, P. , Piraino, A. , Cortesi, P.A. et al. (2024) Cost and cost effectiveness of treatments for psoriatic arthritis: an updated systematic literature review. PharmacoEconomics, 42, 1329–1343. 39182010 10.1007/s40273-024-01428-1

[aos70060-bib-0002] Belotti, F. , Deb, P. , Manning, W.G. & Norton, E.C. (2015) Twopm: two‐part models. The Stata Journal: Promoting Communications on Statistics and Stata, 15, 3–20.

[aos70060-bib-0003] Bourne, R.R.A. , Steinmetz, J.D. , Flaxman, S. , Briant, P.S. , Taylor, H.R. , Resnikoff, S. et al. (2021) Trends in prevalence of blindness and distance and near vision impairment over 30 years: an analysis for the global burden of disease study. The Lancet Global Health, 9, e130–e143.33275950

[aos70060-bib-0004] Chakravarthy, U. , Biundo, E. , Saka, R.O. , Fasser, C. , Bourne, R. & Little, J.A. (2017) The economic impact of blindness in Europe. Ophthalmic Epidemiology, 24, 239–247. 28665742 10.1080/09286586.2017.1281426

[aos70060-bib-0005] Chuvarayan, Y. , Finger, R.P. & Köberlein‐Neu, J. (2020) Economic burden of blindness and visual impairment in Germany from a societal perspective: a cost‐of‐illness study. The European Journal of Health Economics, 21, 115–127. 31493181 10.1007/s10198-019-01115-5

[aos70060-bib-0006] Finnish Centre for Pensions . (2012) Statistical yearbook of pensioners in Finland 2011 (official statistics of Finland). Helsingfors: Eläketurvakeskus.

[aos70060-bib-0007] Finnish Institute for Health and Welfare . (2024) Sotkanet Database. Helsinki: Finnish Institute for Health and Welfare.

[aos70060-bib-0008] Frick, K.D. , Walt, J.G. , Chiang, T.H. , Doyle, J.J. , Stern, L.S. , Katz, L.M. et al. (2008) Direct costs of blindness experienced by patients enrolled in managed care. Ophthalmology, 115, 11–17. 17475331 10.1016/j.ophtha.2007.02.007

[aos70060-bib-0009] Frick, K.D. , Roebuck, M.C. , Feldstein, J.I. , McCarty, C.A. & Grover, L.L. (2012) Health services utilization and cost of retinitis pigmentosa. Archives of Ophthalmology, 130, 629–634. 22652848 10.1001/archophthalmol.2011.2820

[aos70060-bib-0010] Gordois, A. , Cutler, H. , Pezzullo, L. , Gordon, K. , Cruess, A. , Winyard, S. et al. (2012) An estimation of the worldwide economic and health burden of visual impairment. Global Public Health, 7, 465–481. 22136197 10.1080/17441692.2011.634815

[aos70060-bib-0011] Green, D. , Ducorroy, G. , McElnea, E. , Naughton, A. , Skelly, A. , O'Neill, C. et al. (2016) The cost of blindness in the Republic of Ireland 2010‐2020. Journal of Ophthalmology, 2016, 1–8. 10.1155/2016/4691276 PMC4770135 26981276

[aos70060-bib-0012] Heistaro, S. (2008) Methodology report : health 2000 survey (No. B26/2008). Helsinki: Publications of the National Public Health Institute.

[aos70060-bib-0013] Husereau, D. , Drummond, M. , Augustovski, F. , de Bekker‐Grob, E. , Briggs, A.H. , Carswell, C. et al. (2022) Consolidated health economic evaluation reporting standards (CHEERS) 2022 explanation and elaboration: a report of the ISPOR CHEERS II good practices task force. Value in Health, 25, 10–31. 35031088 10.1016/j.jval.2021.10.008

[aos70060-bib-0014] Järnefelt, N. , Kautto, M. , Nurminen, M. & Salonen, J. (2013) Työurien pituuden kehitys 2000‐luvulla (No. 01/2013). Helsinki: Eläketurvakeskuksen Raportteja.

[aos70060-bib-0015] Jin, H. & McCrone, P. (2015) Cost‐of‐illness studies for bipolar disorder: systematic review of international studies. PharmacoEconomics, 33, 341–353. 25576148 10.1007/s40273-014-0250-y

[aos70060-bib-0016] Jo, C. (2014) Cost‐of‐illness studies: concepts, scopes, and methods. Clinical and Molecular Hepatology, 20, 327–337. 25548737 10.3350/cmh.2014.20.4.327 PMC4278062

[aos70060-bib-0017] Kapiainen, S. , Väisänen, A. & Haula, T. (2014) Terveyden‐ja sosiaalihuollon yksikkökustannukset Suomessa vuonna 2011 (No. 3/2014). Helsinki: Publications of the Finnish National Institute for Health and Welfare.

[aos70060-bib-0018] Köberlein, J. , Beifus, K. , Schaffert, C. & Finger, R.P. (2013) The economic burden of visual impairment and blindness: a systematic review. BMJ Open, 3, 1258. 10.1136/bmjopen-2013-003471 PMC3822298 24202057

[aos70060-bib-0019] Koskinen, S. , Lundqvist, A. & Ristiluoma, N. (2012) Health, functional capacity and welfare in Finland in 2011 (No. 68/2012). Helsinki: Publications of the Finnish National Institute for Health and Welfare.

[aos70060-bib-0020] Kurz, C.F. (2017) Tweedie distributions for fitting semicontinuous health care utilization cost data. BMC Medical Research Methodology, 17, 171. 29258428 10.1186/s12874-017-0445-y PMC5735804

[aos70060-bib-0021] Kuusisto, T. (Ed.). (2019) Official statistics of Finland (OSF): annual national accounts. Helsinki: Statistics Finland.

[aos70060-bib-0022] Leeper, T.J. (2021) margins: Marginal effects for model objects.

[aos70060-bib-0023] Liang, J. , Pu, Y. , Chen, J. , Liu, M. , Ouyang, B. , Jin, Z. et al. (2025) Global prevalence, trend and projection of myopia in children and adolescents from 1990 to 2050: a comprehensive systematic review and meta‐analysis. The British Journal of Ophthalmology, 109, 362–371. 39317432 10.1136/bjo-2024-325427

[aos70060-bib-0024] Lumley, T. (2004) Analysis of complex survey samples. Journal of Statistical Software, 9, 1–19.

[aos70060-bib-0025] Lundqvist, A. & Mäki‐Opas, T. (2016) Health 2011 survey ‐ methods (No. 8/2016). Helsinki: Publications of the Finnish National Institute for health and welfare.

[aos70060-bib-0026] Marques, A.P. , Macedo, A.F. , Hernandez‐Moreno, L. , Ramos, P.L. , Butt, T. , Rubin, G. et al. (2018) The use of informal care by people with vision impairment. PLoS One, 13, e0198631. 29879193 10.1371/journal.pone.0198631 PMC5991749

[aos70060-bib-0027] Marques, A.P. , Ramke, J. , Cairns, J. , Butt, T. , Zhang, J.H. , Muirhead, D. et al. (2021) Global economic productivity losses from vision impairment and blindness. EClinicalMedicine, 35, 100852. 33997744 10.1016/j.eclinm.2021.100852 PMC8093883

[aos70060-bib-0028] Matveinen, P. (2021) Health expenditure and financing 2019 (C2 No. 15/2021). Helsinki: Finnish Institute for Health and Welfare.

[aos70060-bib-0029] Mihaylova, B. , Briggs, A. , O'Hagan, A. & Thompson, S.G. (2011) Review of statistical methods for analysing healthcare resources and costs. Health Economics, 20, 897–916. 20799344 10.1002/hec.1653 PMC3470917

[aos70060-bib-0030] Morse, A.R. , Yatzkan, E. , Berberich, B. & Arons, R.R. (1999) Acute care hospital utilization by patients with visual impairment. Archives of Ophthalmology, 117, 943–949. 10408461 10.1001/archopht.117.7.943

[aos70060-bib-0031] Morse, A.R. , Seiple, W. , Talwar, N. , Lee, P.P. & Stein, J.D. (2019) Association of Vision Loss with hospital use and costs among older adults (vol. 137). In: Presented at the JAMA ophthalmology. Chicago, IL: American Medical Association, pp. 634–640. 10.1001/jamaophthalmol.2019.0446 PMC6567836 30946451

[aos70060-bib-0032] Pablo, L. , Garay‐Aramburu, G. , García Layana, A. , Fernandez, A. , Vázquez, I. , Acebes, X. et al. (2024) Assessing the economic burden of vision loss and irreversible legal blindness in Spain (2021–2030): a societal perspective. Health Economics Review, 14, 70. 39225974 10.1186/s13561-024-00546-y PMC11370269

[aos70060-bib-0033] Pezzullo, L. , Streatfeild, J. , Simkiss, P. & Shickle, D. (2018) The economic impact of sight loss and blindness in the UK adult population. BMC Health Services Research, 18, 63. 29382329 10.1186/s12913-018-2836-0 PMC5791217

[aos70060-bib-0034] Purola, P. , Ojamo, M.U.I. , Gissler, M. & Uusitalo, H.M.T. (2022) Changes in visual impairment due to diabetic retinopathy during 1980–2019 based on Nationwide register data. Diabetes Care, 45, 2020–2027. 35838317 10.2337/dc21-2369 PMC9472510

[aos70060-bib-0035] Purola, P. , Kaarniranta, K. , Ojamo, M. , Gissler, M. & Uusitalo, H. (2023) Visual impairment due to age‐related macular degeneration during 40 years in Finland and the impact of novel therapies. Acta Ophthalmologica, 101, 57–64. 35912685 10.1111/aos.15224 PMC10087211

[aos70060-bib-0036] Purola, P. , Taipale, J. , Väätäinen, S. , Harju, M. , Koskinen, S.V.P. & Uusitalo, H.M.T. (2023) Price tag of glaucoma care is minor compared with the total direct and indirect costs of glaucoma: results from nationwide survey and register data. PLoS One, 18, e0295523. 38117760 10.1371/journal.pone.0295523 PMC10732367

[aos70060-bib-0037] Rein, D.B. , Wittenborn, J.S. , Zhang, P. , Sublett, F. , Lamuda, P.A. , Lundeen, E.A. et al. (2021) The economic burden of vision loss and blindness in the United States. Ophthalmology, 25, 158. 10.1016/j.ophtha.2021.09.010 34560128

[aos70060-bib-0038] Sonron, E.‐A. , Tripathi, V. & Hariharan, S. (2020) The impact of sociodemographic and socioeconomic factors on the burden of cataract in Small Island developing states (SIDS) in the Caribbean from 1990 to 2016. Ophthalmic Epidemiology, 27, 132–140. 31818167 10.1080/09286586.2019.1700534

[aos70060-bib-0039] Statistics Finland . (2023) Official statistics of Finland (OSF): population structure [e‐publication]. Off stat Finl OSF Popul struct E‐Publ Annu rev 2011. Helsinki, Finland: Statistics Finland.

[aos70060-bib-0040] Statistics Finland . (2024) Official statistics of Finland (OSF): consumer price index [online publication]. Helsinki, Finland: Statistics Finland.

[aos70060-bib-0041] Taipale, J. , Mikhailova, A. , Ojamo, M. , Nättinen, J. , Väätäinen, S. , Gissler, M. et al. (2019) Low vision status and declining vision decrease health‐related quality of life: results from a nationwide 11‐year follow‐up study. Quality of Life Research, 28, 3225–3236. 31401749 10.1007/s11136-019-02260-3 PMC6863947

[aos70060-bib-0042] Taipale, J. , Purola, P.K.M. , Väätäinen, S. , Nättinen, J.E. , Koskinen, S.V.P. & Uusitalo, H.M.T. (2025) Societal costs of decreased visual acuity: a Finnish cohort study with 15 years of registry data follow‐up. Acta Ophthalmologica, 25, 17439. 10.1111/aos.17439 39831590

[aos70060-bib-0043] Tevameri, T. (2018) Sector reports – health and social services, from an uncertain outlook to increased wellbeing? (No. 38/2018). Helsinki: Ministry of Economic Affairs and Employment.

[aos70060-bib-0044] Tham, Y.‐C. , Li, X. , Wong, T.Y. , Quigley, H.A. , Aung, T. & Cheng, C.‐Y. (2014) Global prevalence of glaucoma and projections of glaucoma burden through 2040: a systematic review and meta‐analysis. Ophthalmology, 121, 2081–2090. 24974815 10.1016/j.ophtha.2014.05.013

[aos70060-bib-0045] The Social Insurance Institution of Finland . (2023) Statistical Database Kelasto. Helsinki: The Social Insurance Institution of Finland.

[aos70060-bib-0046] Tsang, J.Y. , Wright, A. , Carr, M.J. , Dickinson, C. , Harper, R.A. , Kontopantelis, E. et al. (2024) Risk of falls and fractures in individuals with cataract, age‐related macular degeneration, or glaucoma. JAMA Ophthalmology, 142, 96–106. 38153708 10.1001/jamaophthalmol.2023.5858 PMC10870181

[aos70060-bib-0047] Vaajanen, A. , Purola, P. , Ojamo, M. , Gissler, M. & Uusitalo, H. (2022) Changes in incidence and severity of visual impairment due to glaucoma during 40 years – a register‐based study in Finland. Acta Ophthalmologica, 100, 534–540. 34595821 10.1111/aos.15030

[aos70060-bib-0048] Väätäinen, S. , Soini, E. , Arvonen, S. , Suojanen, L. & Pietiläinen, K. (2019) Potential direct secondary care cost benefits of HealthyWeightHub – virtual hospital 2.0 digital lifestyle intervention. Finnish Journal of EHealth and EWelfare, 11, 342–356.

[aos70060-bib-0049] Von Elm, E. , Altman, D.G. , Egger, M. , Pocock, S.J. , Gøtzsche, P.C. , Vandenbroucke, J.P. et al. (2007) The strengthening the reporting of observational studies in epidemiology (STROBE) statement: guidelines for reporting observational studies. Annals of Internal Medicine, 147, 573. 17938396 10.7326/0003-4819-147-8-200710160-00010

[aos70060-bib-0050] Wong, W.L. , Su, X. , Li, X. , Cheung, C.M.G. , Klein, R. , Cheng, C.‐Y. et al. (2014) Global prevalence of age‐related macular degeneration and disease burden projection for 2020 and 2040: a systematic review and meta‐analysis. The Lancet Global Health, 2, e106–e116. 25104651 10.1016/S2214-109X(13)70145-1

[aos70060-bib-0051] Xu, Y. , Wang, A. , Lin, X. , Xu, J. , Shan, Y. , Pan, X. et al. (2021) Global burden and gender disparity of vision loss associated with diabetes retinopathy. Acta Ophthalmologica, 99, 431–440. 33124190 10.1111/aos.14644

[aos70060-bib-0052] Zhang, J. , Wu, Y. , Sharma, B. , Gupta, R. , Jawla, S. & Bullimore, M.A. (2023) Epidemiology and burden of astigmatism: a systematic literature review. Optometry and Vision Science, 100, 218–231. 36749017 10.1097/OPX.0000000000001998 PMC10045990

