# Supplementary material for: Direct and indirect costs associated with declining distance visual acuity: A nationwide longitudinal cost‐of‐illness study with 11‐year follow‐up
Source: Acta Ophthalmol. 2026 Jan 27;104(5):559–71. doi: 10.1111/aos.70060 (PMC13353656; doi:10.1111/aos.70060)
Supplement: Supplementary file 1 — Tables S1–S10. [file AOS-104-559-s002.docx]

**Table S1. Multivariable two-part regression analysis examining the impact of declining visual acuity, age, sex, and non-eye-related comorbidities on average annual costs of inpatient care (primary, secondary, and psychiatric) at the 2019 cost level.**

|  | **Model B coefficients (p-values)** | | **Average Marginal Effects (AME) in euros** | | |
| --- | --- | --- | --- | --- | --- |
|  | **First part** | **Second part** | **Mean AME contrast** | **Standard Error (SE)** | **P value** |
| Constant/ Intercept | 1.16 (0.207) | 7.51 (<0.001) |  |  |  |
| Age at baseline, per year | 0.02 (<0.001) | 0.01 (<0.001) | **11.01** | **2.19** | **<0.001** |
| Male sex | -0.34 (<0.001) | 0.03 (0.715) | -47.87 | 40.39 | 0.236 |
| The level of education, low (1) | Reference | Reference | Reference |  |  |
| The level of education, middle (2) | -0.01 (0.940) | -0.13 (0.176) | -65.82 | 50.28 | 0.191 |
| The level of education, high (3) | -0.11 (0.286) | -0.25 (0.012) | **-143.93** | **50.93** | **0.005** |
| Incident heart disease | 0.94 (<0.001) | 0.37 (<0.001) | **411.94** | **81.76** | **<0.001** |
| Incident pulmonary disease | 0.76 (<0.001) | 0.61 (<0.001) | **610.24** | **130.15** | **<0.001** |
| Incident vascular disease | 0.54 (0.004) | 0.53 (<0.001) | **485.85** | **139.72** | **<0.001** |
| Incident musculoskeletal condition | 0.30 (<0.001) | 0.11 (0.149) | **113.38** | **45.13** | **0.012** |
| Incident hypertension | 0.31 (0.008) | -0.10 (0.332) | 4.57 | 53.20 | 0.932 |
| Incident diabetes | 0.43 (0.012) | 0.27 (0.038) | **249.40** | **101.59** | **0.014** |
| Incident psychiatric disorder | 0.72 (<0.001) | 0.24 (0.146) | **282.90** | **133.43** | **0.034** |
| Incident cancer | 1.08 (<0.001) | 0.26 (0.061) | **356.79** | **121.77** | **0.003** |
| Baseline distance VA * | -0.17 (0.017) | -0.14 (0.004) | **-101.08** | **28.41** | **<0.001** |
| Declining distance VA | 0.50 (0.004) | 0.79 (<0.001) | **718.94** | **152.24** | **<0.001** |
| Improving distance VA | -0.08 (0.811) | -0.35 (0.152) | -160.51 | 96.39 | 0.096 |

Logistic regression model was used for the first part, and generalized linear model with Gamma distribution and log link for the second part. The analysis was based on participants with information available for all predictors (*n* = 3,803). The age and other predictors were standardized at the mean level of those with non-declining (including those with improving) VA (n=3,512) for calculating the AMEs, which were compared to the observed mean in the non-declining VA group (447.39 €). AME contrasts equal the difference between those with a medical condition (or of male sex) and those without a medical condition (or of female sex), standardized for all other factors. Bolded contrasts denote statistically significant differences (p < 0.05).

* The number of correctly identified eye-chart lines from VA 0.0 to VA 1.0. The mean AME contrast of
-101.08 € corresponds to the cost reduction when correctly identifying one more eye-chart line in the baseline, up to VA 1.0.

**Table S2. Multivariable regression analysis examining the impact of declining visual acuity, age, sex, and non-eye-related comorbidities on average annual costs of all secondary outpatient care at the 2019 cost level.**

|  | **Model B coefficients** | | **Average Marginal Effects (AME) in euros** | | |
| --- | --- | --- | --- | --- | --- |
|  | **B coefficient** | **P value** | **Mean AME contrast** | **Standard Error (SE)** | **P value** |
| Constant/ Intercept | 6.33 | <0.001 |  |  |  |
| Age at baseline, per year | 0.00 | 0.105 | 1.40 | 0.87 | 0.107 |
| Male sex | -0.36 | <0.001 | **-109.34** | **17.18** | **<0.001** |
| The level of education, low (1) | Reference | Reference | Reference |  |  |
| The level of education, middle (2) | -0.01 | 0.909 | -2.56 | 22.43 | 0.909 |
| The level of education, high (3) | -0.03 | 0.695 | -9.02 | 22.93 | 0.694 |
| Incident heart disease | 0.55 | <0.001 | **208.50** | **37.70** | **<0.001** |
| Incident pulmonary disease | 0.47 | <0.001 | **179.25** | **46.17** | **<0.001** |
| Incident vascular disease | 0.40 | <0.001 | **150.22** | **50.92** | **0.003** |
| Incident musculoskeletal condition | 0.28 | <0.001 | **89.56** | **20.38** | **<0.001** |
| Incident hypertension | 0.16 | 0.037 | **52.81** | **26.91** | **0.050** |
| Incident diabetes | 0.40 | <0.001 | **286.85** | **79.06** | **<0.001** |
| Incident psychiatric disorder | 0.67 | <0.001 | **145.65** | **47.26** | **0.002** |
| Incident cancer | 0.88 | <0.001 | **413.95** | **84.90** | **<0.001** |
| Baseline distance VA * | -0.09 | 0.030 | **-26.79** | **12.46** | **0.032** |
| Declining distance VA | 0.39 | <0.001 | **140.21** | **44.94** | **0.002** |
| Improving distance VA | -0.25 | 0.203 | -69.02 | 48.11 | 0.151 |

Tweedie family with gamma distribution and log link scale response was applied to the regression model. The analysis was based on participants with information available for all predictors (*n* = 3,803). The age and other predictors were standardized at the mean level of those with non-declining (including those with improving) VA (n=3,512) for calculating the AMEs, which were compared to the observed mean in the non-declining VA group (277.74 €). AME contrasts equal the difference between those with a medical condition (or of male sex) and those without a medical condition (or of female sex), standardized for all other factors. Bolded contrasts denote statistically significant differences (p < 0.05).

* The number of correctly identified eye-chart lines from VA 0.0 to VA 1.0. The mean AME contrast of
-26.79 € corresponds to the cost reduction when correctly identifying one more eye-chart line in the baseline, up to VA 1.0.

**Table S3. Multivariable two-part regression analysis examining the impact of declining visual acuity, age, sex, and non-eye-related comorbidities on average annual costs of eye-related secondary outpatient care at the 2019 cost level.**

|  | **Model B coefficients (p-values)** | | **Average Marginal Effects (AME) in euros** | | |
| --- | --- | --- | --- | --- | --- |
|  | **First part** | **Second part** | **Mean AME contrast** | **Standard Error (SE)** | **P value** |
| Constant/ Intercept | -0.48 (0.578) | 4.62 (<0.001) |  |  |  |
| Age at baseline, per year | 0.06 (<0.001) | 0.02 (<0.001) | **0.85** | **0.11** | **<0.001** |
| Male sex | -0.23 (0.015) | 0.16 (0.100) | -0.32 | 1.40 | 0.816 |
| The level of education, low (1) | Reference | Reference | Reference |  |  |
| The level of education, middle (2) | 0.11 (0.386) | 0.03 (0.834) | 1.26 | 1.80 | 0.483 |
| The level of education, high (3) | 0.26 (0.039) | 0.11 (0.371) | 3.76 | 1.99 | 0.059 |
| Incident heart disease | 0.37 (0.002) | -0.07 (0.522) | 2.76 | 1.91 | 0.148 |
| Incident pulmonary disease | 0.18 (0.248) | -0.16 (0.296) | -0.16 | 2.21 | 0.944 |
| Incident vascular disease | 0.19 (0.284) | 0.04 (0.828) | 2.32 | 2.92 | 0.426 |
| Incident musculoskeletal condition | 0.21 (0.030) | 0.03 (0.784) | 2.30 | 1.50 | 0.126 |
| Incident hypertension | 0.06 (0.624) | 0.13 (0.284) | 2.18 | 2.05 | 0.286 |
| Incident diabetes | 0.35 (0.033) | -0.09 (0.573) | 2.30 | 2.59 | 0.375 |
| Incident psychiatric disorder | -0.20 (0.428) | 0.12 (0.647) | -0.50 | 3.65 | 0.891 |
| Incident cancer | -0.07 (0.707) | -0.07 (0.699) | -1.34 | 2.37 | 0.570 |
| Baseline distance VA * | -0.34 (<0.001) | -0.12 (0.004) | **-4.48** | **0.85** | **<0.001** |
| Declining distance VA | 0.15 (0.338) | 0.49 (<0.001) | **8.69** | **3.51** | **0.013** |
| Improving distance VA | -0.37 (0.218) | -0.82 (<0.001) | **-7.66** | **1.50** | **<0.001** |

Logistic regression model was used for the first part, and generalized linear model with Gamma distribution and log link for the second part. The analysis was based on participants with information available for all predictors (*n* = 3,803). The age and other predictors were standardized at the mean level of those with non-declining (including those with improving) VA (n=3,512) for calculating the AMEs, which were compared to the observed mean in the non-declining VA group (11.42 €). AME contrasts equal the difference between those with a medical condition (or of male sex) and those without a medical condition (or of female sex), standardized for all other factors. Bolded contrasts denote statistically significant differences (p < 0.05).

* The number of correctly identified eye-chart lines from VA 0.0 to VA 1.0. The mean AME contrast of
-4.48 € corresponds to the cost reduction when correctly identifying one more eye-chart line in the baseline, up to VA 1.0.

**Table S4. Multivariable two-part regression analysis examining the impact of declining visual acuity, age, sex, and non-eye-related comorbidities on average annual costs of primary, occupational and private care physician visits at the 2019 cost level.**

|  | **Model B coefficients (p-values)** | | **Average Marginal Effects (AME) in euros** | | |
| --- | --- | --- | --- | --- | --- |
|  | **First part** | **Second part** | **Mean AME contrast** | **Standard Error (SE)** | **P value** |
| Constant/ Intercept | -0.40 (0.846) | 6.87 (<0.001) |  |  |  |
| Age at baseline, per year | -0.11 (<0.001) | -0.01 (0.055) | **-8.89** | **1.24** | **<0.001** |
| Male sex | -0.44 (<0.001) | -0.37 (<0.001) | **-84.92** | **12.55** | **<0.001** |
| The level of education, low (1) | Reference | Reference | Reference |  |  |
| The level of education, middle (2) | 0.02 (0.906) | -0.23 (0.025) | -29.72 | 19.68 | 0.131 |
| The level of education, high (3) | 0.18 (0.364) | -0.19 (0.056) | -13.80 | 20.01 | 0.490 |
| Incident heart disease | 0.29 (0.242) | 0.33 (0.005) | **83.68** | **35.46** | **0.018** |
| Incident pulmonary disease | 0.28 (0.235) | 0.29 (0.009) | **73.15** | **31.70** | **0.021** |
| Incident vascular disease | 0.93 (0.004) | 0.03 (0.809) | **72.42** | **32.94** | **0.028** |
| Incident musculoskeletal condition | 0.48 (<0.001) | 0.06 (0.356) | **44.30** | **14.38** | **0.002** |
| Incident hypertension | 0.57 (0.002) | 0.24 (0.004) | **87.36** | **23.68** | **<0.001** |
| Incident diabetes | 0.34 (0.286) | 0.41 (0.007) | **107.93** | **50.60** | **0.033** |
| Incident psychiatric disorder | 0.47 (0.094) | 0.39 (0.001) | **117.00** | **41.90** | **0.005** |
| Incident cancer | 0.04 (0.914) | -0.27 (0.139) | -31.58 | 29.56 | 0.285 |
| Baseline distance VA * | 0.37 (0.029) | -0.03 (0.745) | 21.97 | 19.00 | 0.248 |
| Declining distance VA | -0.80 (0.037) | 0.26 (0.250) | -28.73 | 40.29 | 0.476 |
| Improving distance VA | 0.65 (0.358) | 0.38 (0.377) | 132.21 | 134.14 | 0.324 |

Logistic regression model was used for the first part, and generalized linear model with Gamma distribution and log link for the second part. The analysis was based on participants with information available for all predictors (*n* = 1,650). The age and other predictors were standardized at the mean level of those with non-declining (including those with improving) VA (n=1,514) for calculating the AMEs, which were compared to the observed mean in the non-declining VA group (142.05 €). AME contrasts equal the difference between those with a medical condition (or of male sex) and those without a medical condition (or of female sex), standardized for all other factors. Bolded contrasts denote statistically significant differences (p < 0.05).

* The number of correctly identified eye-chart lines from VA 0.0 to VA 1.0. The mean AME contrast of
21.97 € corresponds to the cost increase when correctly identifying one more eye-chart line in the baseline, up to VA 1.0.

**Table S5. Multivariable two-part regression analysis examining the impact of declining visual acuity, age, sex, and non-eye-related comorbidities on average annual costs of primary, occupational and private care nurse visits at the 2019 cost level.**

|  | **Model B coefficients (p-values)** | | **Average Marginal Effects (AME) in euros** | | |
| --- | --- | --- | --- | --- | --- |
|  | **First part** | **Second part** | **Mean AME contrast** | **Standard Error (SE)** | **P value** |
| Constant/ Intercept | 0.52 (0.676) | 8.16 (<0.001) |  |  |  |
| Age at baseline, per year | 0.00 (0.784) | 0.03 (<0.001) | **2.25** | **0.80** | **0.005** |
| Male sex | 0.02 (0.879) | -0.39 (0.012) | **-22.54** | **10.10** | **0.026** |
| The level of education, low (1) | Reference | Reference | Reference |  |  |
| The level of education, middle (2) | -0.02 (0.928) | -0.27 (0.251) | -16.26 | 14.59 | 0.265 |
| The level of education, high (3) | 0.02 (0.925) | -0.38 (0.121) | -21.61 | 15.40 | 0.160 |
| Incident heart disease | 0.35 (0.082) | 0.63 (0.016) | **71.39** | **34.42** | **0.038** |
| Incident pulmonary disease | 0.12 (0.573) | 0.83 (0.004) | **81.45** | **41.01** | **0.047** |
| Incident vascular disease | 0.02 (0.948) | 0.12 (0.729) | 8.10 | 24.89 | 0.745 |
| Incident musculoskeletal condition | 0.06 (0.610) | 0.01 (0.954) | 2.74 | 10.94 | 0.802 |
| Incident hypertension | 0.58 (<0.001) | 0.16 (0.405) | 33.62 | 17.85 | 0.060 |
| Incident diabetes | 1.13 (<0.001) | 0.96 (0.001) | **187.38** | **72.82** | **0.010** |
| Incident psychiatric disorder | 0.14 (0.581) | 0.23 (0.505) | 21.02 | 28.94 | 0.468 |
| Incident cancer | 0.11 (0.709) | -0.47 (0.225) | -20.06 | 17.25 | 0.245 |
| Baseline distance VA * | -0.08 (0.397) | -0.36 (0.002) | **-25.79** | **9.60** | **0.007** |
| Declining distance VA | -0.26 (0.290) | 0.48 (0.171) | 21.77 | 30.12 | 0.470 |
| Improving distance VA | 0.27 (0.596) | -1.41 (0.019) | **-43.29** | **12.34** | **<0.001** |

Logistic regression model was used for the first part, and generalized linear model with Gamma distribution and log link for the second part. The analysis was based on participants with information available for all predictors (*n* = 1,650). The age and other predictors were standardized at the mean level of those with non-declining (including those with improving) VA (n=1,514) for calculating the AMEs, which were compared to the observed mean in the non-declining VA group (85.82 €). AME contrasts equal the difference between those with a medical condition (or of male sex) and those without a medical condition (or of female sex), standardized for all other factors. Bolded contrasts denote statistically significant differences (p < 0.05).

* The number of correctly identified eye-chart lines from VA 0.0 to VA 1.0. The mean AME contrast of
-25.79 € corresponds to the cost reduction when correctly identifying one more eye-chart line in the baseline, up to VA 1.0.

**Table S6. Multivariable regression analysis examining the impact of declining visual acuity, age, sex, and non-eye-related comorbidities on average annual outpatient travel expenses at the 2019 cost level.**

|  | **Model B coefficients** | | **Average Marginal Effects (AME) in euros** | | |
| --- | --- | --- | --- | --- | --- |
|  | **B coefficient** | **P value** | **Mean AME contrast** | **Standard Error (SE)** | **P value** |
| Constant/ Intercept | 4.12 | <0.001 |  |  |  |
| Age at baseline, per year | 0.01 | 0.073 | 0.13 | 0.07 | 0.075 |
| Male sex | -0.34 | <0.001 | -8.58 | 1.44 | 0.893 |
| The level of education, low (1) | Reference | Reference | Reference |  |  |
| The level of education, middle (2) | -0.01 | 0.893 | **-0.25** | **1.89** | **<0.001** |
| The level of education, high (3) | -0.06 | 0.404 | **-1.61** | **1.91** | **0.008** |
| Incident heart disease | 0.52 | <0.001 | 15.94 | 3.10 | 0.106 |
| Incident pulmonary disease | 0.51 | <0.001 | **16.22** | **4.03** | **0.002** |
| Incident vascular disease | 0.37 | 0.002 | **11.00** | **4.15** | **0.007** |
| Incident musculoskeletal condition | 0.26 | <0.001 | **6.86** | **1.70** | **0.015** |
| Incident hypertension | 0.14 | 0.089 | **3.59** | **2.22** | **<0.001** |
| Incident diabetes | 0.42 | <0.001 | **20.57** | **6.27** | **<0.001** |
| Incident psychiatric disorder | 0.61 | <0.001 | **12.66** | **4.05** | **<0.001** |
| Incident cancer | 0.85 | <0.001 | **32.27** | **6.96** | **<0.001** |
| Baseline distance VA * | -0.11 | 0.007 | **-2.82** | **1.05** | **<0.001** |
| Declining distance VA | 0.35 | 0.001 | **10.17** | **3.66** | **<0.001** |
| Improving distance VA | -0.41 | 0.046 | **-8.50** | **3.50** | **<0.001** |

Tweedie family with gamma distribution and log link scale response was applied to the regression model. The analysis was based on participants with information available for all predictors (*n* = 3,803). The age and other predictors were standardized at the mean level of those with non-declining (including those with improving) VA (n=3,512) for calculating the AMEs, which were compared to the observed mean in the non-declining VA group (22.65 €). AME contrasts equal the difference between those with a medical condition (or of male sex) and those without a medical condition (or of female sex), standardized for all other factors. Bolded contrasts denote statistically significant differences (p < 0.05).

* The number of correctly identified eye-chart lines from VA 0.0 to VA 1.0. The mean AME contrast of
-2.82 € corresponds to the cost reduction when correctly identifying one more eye-chart line in the baseline, up to VA 1.0.

**Table S7. Multivariable regression analysis examining the impact of declining visual acuity, age, sex, and non-eye-related comorbidities on average annual early retirement pensions at the 2019 cost level among participants aged 30-64 years.**

|  | **Model B coefficients** | | **Average Marginal Effects (AME) in euros** | | |
| --- | --- | --- | --- | --- | --- |
|  | **B coefficient** | **P value** | **Mean AME contrast** | **Standard Error (SE)** | **P value** |
| Constant/ Intercept | 12.14 | <0.001 |  |  |  |
| Age at baseline, per year | -0.05 | <0.001 | **-234.20** | **21.28** | **<0.001** |
| Male sex | -0.01 | 0.884 | -32.98 | 226.42 | 0.884 |
| The level of education, low (1) | Reference | Reference | Reference |  |  |
| The level of education, middle (2) | -0.10 | 0.056 | -490.33 | 251.71 | 0.051 |
| The level of education, high (3) | -0.37 | <0.001 | **-1,636.16** | **240.16** | **<0.001** |
| Incident heart disease | 0.06 | 0.336 | 279.98 | 296.25 | 0.345 |
| Incident pulmonary disease | 0.06 | 0.401 | 314.16 | 383.24 | 0.412 |
| Incident vascular disease | 0.12 | 0.192 | 612.14 | 493.83 | 0.215 |
| Incident musculoskeletal condition | -0.08 | 0.081 | -405.58 | 229.37 | 0.077 |
| Incident hypertension | 0.01 | 0.836 | 61.77 | 300.20 | 0.837 |
| Incident diabetes | 0.24 | 0.001 | **1,306.59** | **424.68** | **0.002** |
| Incident psychiatric disorder | 0.04 | 0.741 | 185.41 | 570.96 | 0.745 |
| Incident cancer | -0.02 | 0.787 | -114.65 | 419.27 | 0.785 |
| Baseline distance VA * | -0.09 | 0.004 | **-419.72** | **145.69** | **0.004** |
| Declining distance VA | 0.41 | <0.001 | **2,355.72** | **530.42** | **<0.001** |
| Improving distance VA | -0.17 | 0.273 | -772.73 | 649.67 | 0.234 |

Tweedie family with gamma distribution and log link scale response was applied to the regression model. The analysis was based on participants aged 30-64 years, with information available for all predictors (*n* = 1,289). The age and other predictors were standardized at the mean level of those with non-declining (including those with improving) VA (n=1,170) for calculating the AMEs, which were compared to the observed mean in the non-declining VA group (4,040.38 €). AME contrasts equal the difference between those with a medical condition (or of male sex) and those without a medical condition (or of female sex), standardized for all other factors. Bolded contrasts denote statistically significant differences (p < 0.05).

* The number of correctly identified eye-chart lines from VA 0.0 to VA 1.0. The mean AME contrast of
-419.72 € corresponds to the cost reduction when correctly identifying one more eye-chart line in the baseline, up to VA 1.0.

**Table S8. Multivariable regression analysis examining the impact of declining visual acuity, age, sex, and non-eye-related comorbidities on average annual productivity losses due to early retirement at the 2019 cost level among participants aged 30-64 years.**

|  | **Model B coefficients** | | **Average Marginal Effects (AME) in euros** | | |
| --- | --- | --- | --- | --- | --- |
|  | **B coefficient** | **P value** | **Mean AME contrast** | **Standard Error (SE)** | **P value** |
| Constant/ Intercept | 12.94 | <0.001 |  |  |  |
| Age at baseline, per year | -0.05 | <0.001 | **-523.88** | **47.59** | **<0.001** |
| Male sex | -0.01 | 0.884 | -73.78 | 506.46 | 0.884 |
| The level of education, low (1) | Reference | Reference | Reference |  |  |
| The level of education, middle (2) | -0.10 | 0.056 | -1,096.77 | 563.02 | 0.051 |
| The level of education, high (3) | -0.37 | <0.001 | **-3,659.74** | **537.19** | **<0.001** |
| Incident heart disease | 0.06 | 0.336 | 626.26 | 662.65 | 0.345 |
| Incident pulmonary disease | 0.06 | 0.401 | 702.84 | 857.23 | 0.412 |
| Incident vascular disease | 0.12 | 0.192 | 1,369.14 | 1,104.59 | 0.215 |
| Incident musculoskeletal condition | -0.08 | 0.081 | -907.23 | 513.05 | 0.077 |
| Incident hypertension | 0.01 | 0.836 | 138.03 | 671.48 | 0.837 |
| Incident diabetes | 0.24 | <0.001 | **2,922.65** | **949.94** | **0.002** |
| Incident psychiatric disorder | 0.04 | 0.741 | 414.70 | 1,277.12 | 0.745 |
| Incident cancer | -0.02 | 0.787 | -256.46 | 937.83 | 0.785 |
| Baseline distance VA * | -0.09 | 0.004 | **-939.13** | **325.89** | **0.004** |
| Declining distance VA | 0.41 | <0.001 | **5,269.16** | **1,186.44** | **<0.001** |
| Improving distance VA | -0.17 | 0.273 | -1,729.32 | 1,453.05 | 0.234 |

Tweedie family with gamma distribution and log link scale response was applied to the regression model. The analysis was based on participants aged 30-64 years, with information available for all predictors (*n* = 1,289). The age and other predictors were standardized at the mean level of those with non-declining (including those with improving) VA (n=1,170) for calculating the AMEs, which were compared to the observed mean in the non-declining VA group (9,037.49 €). AME contrasts equal the difference between those with a medical condition (or of male sex) and those without a medical condition (or of female sex), standardized for all other factors. Bolded contrasts denote statistically significant differences (p < 0.05).

* The number of correctly identified eye-chart lines from VA 0.0 to VA 1.0. The mean AME contrast of
-939.13 € corresponds to the cost reduction when correctly identifying one more eye-chart line in the baseline, up to VA 1.0.

**Table S9. Multivariable two-part regression analysis examining the impact of declining visual acuity, age, sex, and non-eye-related comorbidities on average annual productivity losses due to unemployment or layoff at the 2019 cost level among participants aged 30-64 years.**

|  | **Model B coefficients (p-values)** | | **Average Marginal Effects (AME) in euros** | | |
| --- | --- | --- | --- | --- | --- |
|  | **First part** | **Second part** | **Mean AME contrast** | **Standard Error (SE)** | **P value** |
| Constant/ Intercept | -1.26 (0.455) | 10.11 (<0.001) |  |  |  |
| Age at baseline, per year | -0.05 (<0.001) | 0.04 (<0.001) | -1.78 | 15.96 | 0.911 |
| Male sex | 0.25 (0.031) | -0.07 (0.474) | 257.32 | 255.87 | 0.315 |
| The level of education, low (1) | Reference | Reference | Reference |  |  |
| The level of education, middle (2) | 0.04 (0.784) | -0.18 (0.174) | -257.72 | 329.56 | 0.434 |
| The level of education, high (3) | -0.35 (0.035) | -0.46 (0.001) | **-1,285.05** | **331.72** | **<0.001** |
| Incident heart disease | -0.38 (0.079) | 0.04 (0.822) | -470.14 | 391.13 | 0.229 |
| Incident pulmonary disease | 0.04 (0.845) | 0.40 (0.031) | 933.50 | 672.67 | 0.165 |
| Incident vascular disease | -0.30 (0.303) | 0.19 (0.467) | -125.86 | 616.46 | 0.838 |
| Incident musculoskeletal condition | 0.15 (0.225) | -0.04 (0.731) | 167.82 | 275.54 | 0.542 |
| Incident hypertension | 0.19 (0.228) | -0.06 (0.668) | 199.57 | 381.34 | 0.601 |
| Incident diabetes | -0.39 (0.158) | 0.12 (0.639) | -372.51 | 514.68 | 0.469 |
| Incident psychiatric disorder | 0.40 (0.094) | 0.41 (0.046) | 1,928.62 | 1,016.61 | 0.058 |
| Incident cancer | -0.08 (0.795) | -0.13 (0.642) | -331.58 | 590.54 | 0.574 |
| Baseline distance VA * | 0.14 (0.311) | -0.20 (0.139) | -132.21 | 320.92 | 0.680 |
| Declining distance VA | 0.26 (0.343) | -0.01 (0.972) | 427.49 | 730.48 | 0.558 |
| Improving distance VA | 0.93 (0.060) | -0.49 (0.227) | 537.09 | 1,256.34 | 0.669 |

Logistic regression model was used for the first part, and generalized linear model with Gamma distribution and log link for the second part. The analysis was based on participants aged 30-64 years, with information available for all predictors (*n* = 3,369). The age and other predictors were standardized at the mean level of those with non-declining (including those with improving) VA (n=3,204) for calculating the AMEs, which were compared to the observed mean in the non-declining VA group (1,500.80 €). AME contrasts equal the difference between those with a medical condition (or of male sex) and those without a medical condition (or of female sex), standardized for all other factors. Bolded contrasts denote statistically significant differences (p < 0.05).

* The number of correctly identified eye-chart lines from VA 0.0 to VA 1.25. The mean AME contrast of
-132.21 € corresponds to the cost reduction when correctly identifying one more eye-chart line in the baseline.

**Table S10.** **Estimated annual national level direct and indirect cost, and cost-of-illness associated with declining distance VA.**

|  | **Estimated total costs of those with non-declining distance VA, at the national level, €**  **(SE)**  **(A) *** | **Estimated total costs of those with declining distance VA at the national level, €**  **(SE)**  **(B)** | **Total estimated costs at the national level, €**  **(SE)**  **(A+B)** |
| --- | --- | --- | --- |
| Total primary, secondary, and psychiatric inpatient care | 1,457,579,912 (83,203,707) | 384,797,591 (43,619,439) | 1,842,377,503 (104,615,787) |
| Total secondary outpatient care | 904,860,482 (26,833,341) | 122,456,941 (14,375,257) | 1,027,317,423 (33,662,406) |
| Eye-related secondary outpatient care | 37,211,705 (2,536,695) | 8,783,110 (1,213,746) | 45,994,815 (3,059,473) |
| Physician: Primary, occupational, and private care | 462,809,849 (18,664,447) | 16,709,697 (5,334,200) | 479,519,547 (19,522,721) |
| Nurse: Primary, occupational, and private care | 279,599,388 (45,480,140) | 103,894,185 (40,431,588) | 383,493,573 (65,424,044) |
| Travel expenses | 73,786,116 (2,205,844) | 9,820,319 (1,261,421) | 83,606,435 (2,841,415) |
| **Total annual direct health care costs** | **3,178,635,747 (176,387,480)** | **637,678,734 (105,021,904)** | **3,816,314,481 (226,066,373)** |
| Early retirement pensions | 9,928,394,916 (310,745,521) | 391,168,946 (27,853,265) | 10,319,563,862 (308,836,588) |
| Productivity losses due to early retirement | 22,207,741,122 (695,072,888) | 874,963,355 (62,301,924) | 23,082,704,477 (690,802,940) |
| Productivity losses due to unemployment or layoff | 3,687,896,725 (266,884,360) | 142,042,920 (32,186,409) | 3,829,939,645 (268,410,365) |
| **Total annual indirect costs** | **35,824,032,763 (1,272,702,768)** | **1,408,175,221 (122,341,597)** | **37,232,207,984 (1,268,049,893)** |

* Includes participants whose VA improved over time.

Calculated at the population level in 2011 according to Statistics Finland and the stratified cluster design weights by the Finnish Institute for Health and Welfare. The estimated number of citizens with declining distance VA was 248,217, and 69,269 of them were aged from 30 to 64 years. The costs are presented at 2019 levels.
