# Supplementary material for: Direct and indirect costs associated with declining distance visual acuity: A nationwide longitudinal cost‐of‐illness study with 11‐year follow‐up
Source: Acta Ophthalmol. 2026 Jan 27;104(5):559–71. doi: 10.1111/aos.70060 (PMC13353656; doi:10.1111/aos.70060)
Supplement: Supplementary file 2 — Data S1. [file AOS-104-559-s001.docx]

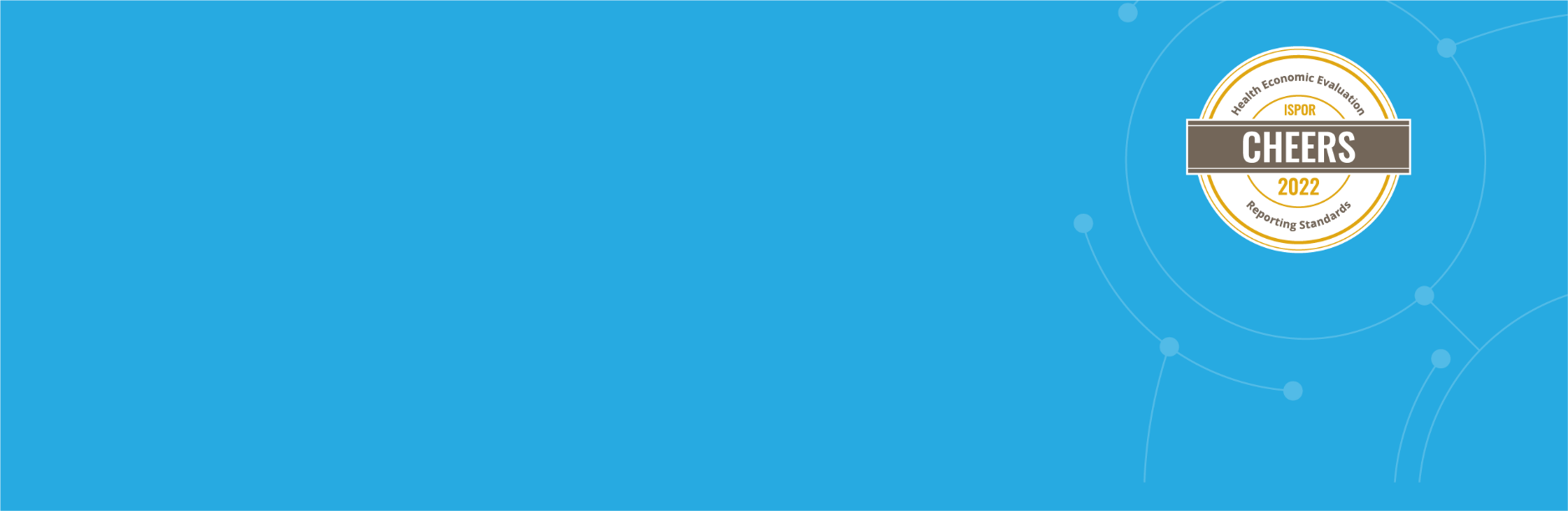


**Consolidated Health Economic Evaluation Reporting Standards
(CHEERS) 2022 Checklist**

The CHEERS 2022 statement replaces the 2013 CHEERS statement, which should no longer
be used. The CHEERS 2022 checklist contains 28 items with accompanying descriptions.
Checklist users should indicate the section of the manuscript where relevant information
can be found. The authors recommend using a section heading with a paragraph number.
If an item does not apply to a particular economic evaluation, checklist users are encouraged
to report “Not Applicable.” If information is otherwise not reported, checklist users are encouraged to

write, “Not Reported.” Users should avoid the term “Not Conducted” as CHEERS is intended to guide and

capture reporting. Additional information on CHEERS 2022 can be found here.

**Title**

**1. Title**

Identify the study as an economic evaluation and specify the interventions being compared.

The direct and indirect costs associated with declining distance visual acuity: A nationwide longitudinal cost-of-illness study with 11-year follow-up.

**Abstract**

**2. Abstract**

Provide a structured summary that highlights context, key methods, results, and alternative analyses.

See Abstract.

**Introduction**

**3. Introduction: Background and Objectives**

Give the context for the study, the study question, and its practical relevance for decision making in policy or practice.

See Introduction.

**Methods**

**4. Health economic analysis plan**

Indicate whether a health economic analysis plan was developed and where available.

Not Applicable.

**5. Study population**

Describe characteristics of the study population (such as age range, demographics, socioeconomic, or clinical characteristics).

The representative sample of the Finnish adult population, based on the Health 2000 Survey and its follow-up study, Health 2011 was examined. The generalizability was improved by utilizing a probability-clustered sampling and weighting scheme. The sample included 3,867 subjects (55% women) aged 30 years and older (mean age 49 years at 2000).

**6. Setting and location**

Provide relevant contextual information that may influence findings.

The target population of the Health 2000 and Health 2011 Surveys consisted of individuals aged 18 or over and living in mainland Finland. In addition to the household population, people living in institutions were included. The main survey was carried out in the population aged 30 or over, and a study of young adults was focused on people aged 18–29. Furthermore, in order to obtain a sufficient number of observations from the oldest age cohorts in the main survey, people aged 80 or over were oversampled using a double sampling fraction.

**7. Comparators**

Describe the interventions or strategies being compared and why chosen.

We conducted a general comparison based on the longitudinal changes in distance visual acuity (VA) groups. VA was measured by a specifically trained study nurse at both time points. Grouping was done according to our previous publication. Declining distance VA was defined as a loss of at least two lines in the logMAR vision chart, equaling 10 ETDRS letters, between the baseline and follow-up examinations. Non-declined VA was included as a comparator group, consisting of those with stable and improved distance VA. Specified direct and indirect healthcare costs were estimated.

**8. Perspective**

State the perspective(s) adopted by the study and why chosen.

There is a need for a comprehensive picture of the economic burden of longitudinally declining visual acuity (VA) including all associated eye- and non-eye-related direct and indirect costs —for example, hospitalizations due to falls and injuries. Additional population-wide studies are also required to corroborate the previous findings and to provide accurate estimates of the costs in different nationwide settings. Moreover, the integration of various data sources, such as national surveys and registers, remains rare, despite its potential to yield more precise estimates on health service usage and both direct and indirect costs. Additionally, current nationwide estimates primarily concentrate on the costs related to visual impairment derived from self-reported survey data, and temporal aspects of declining VA have not currenly been reported. Therefore, the aim of our study was to assess the economic impact of longitudinally declining distance VA, utilizing measured habitual distance VA instead of self-reported VA, within Finnish society and to compare the cost magnitudes of declining and non-declining VA groups. This was accomplished by combining data from nationwide health examination surveys and national health registries, capturing both direct and indirect costs. Our approach also facilitates comparisons of cost impacts with other variables included in the regression models.

**9. Time horizon**

State the time horizon for the study and why appropriate.

The 11-year follow-up perspective is based on the survey setting. Baseline examinations were carried out in 2000, and the follow-up in 2011. To more accurately capture average annual costs, the register-based costs were estimated based on 15 years data from 1.1.1999 to 31.12.2013. This time set was chosen based on the availability of various register data. Furthermore, the long follow-up duration should alleviate the potential biases of long-term consequences in our prevalence-based approach.

**10. Discount rate**

Report the discount rate(s) and reason chosen.

Not Applicable.

**11. Selection of outcomes**

Describe what outcomes were used as the measure(s) of benefit(s) and harm(s).

Not Applicable, as this is a register-based study.

**12. Measurement of outcomes**

Describe how outcomes used to capture benefit(s) and harm(s) were measured.

Not Applicable, as this is a register-based study.

**13. Valuation of outcomes**

Describe the population and methods used to measure and value outcomes.

See Methods: Study population and survey design, Methods: Healthcare utilization, Methods: Healthcare costs, Methods: Indirect costs, and Methods: Data analysis and calculation of population-level estimates.

**14. Measurement and valuation of resources and costs**

Describe how costs were valued.

See Methods: Healthcare utilization, Methods: Healthcare costs, and Methods: Indirect costs.

**15. Currency, price date, and conversion**

Report the dates of the estimated resource quantities and unit costs, plus the currency and year of conversion.

The unit costs are in 2011 level. Travel costs are from previous report in 2017 level. Self-reported doctor and nurse visit quantities and lost working years according to 2011 survey. All the costs are reported in 2019 level for integrity, converted from 2011or 2017 based on the most recent estimates on health expenditure and financing in Finland.

**16. Rationale and description of model**

If modeling is used, describe in detail and why used. Report if the model is publicly available and where it can be accessed.

See Methods: Data analysis and calculation of population-level estimates.

**17. Analytics and assumptions**

Describe any methods for analyzing or statistically transforming data, any extrapolation methods, and approaches for validating any model used.

Because the cost data were right-skewed, maximum likelihood generalized linear modelling was applied using Tweedie with log link scale response for multivariable regression analyses when the zero proportion was under 20%. For those analyses where the zero-proportions were 20% or more, two-step general linear modeling with logistic regression and gamma with log link were applied instead.

**18. Characterizing heterogeneity**

Describe any methods used for estimating how the results of the study vary for subgroups.

We calculated age-, sex- and comorbidity-adjusted results to account for the differences for participants with declining and non-declining distance VA. The results are shown separately for each of the VA groups. In addition, various subpopulation samples were evaluated to evaluate the robustness of the findings across different subsets.

**19. Characterizing distributional effects**

Describe how impacts are distributed across different individuals or adjustments made to reflect priority populations.

Not Applicable.

**20. Characterizing uncertainty**

Describe methods to characterize any sources of uncertainty in the analysis.

To measure the uncertainty, we calculated 95% confidence intervals and standard errors for the results.

**21. Approach to engagement with patients and others affected by the study**

Describe any approaches to engage patients or service recipients, the general public, communities, or stakeholders (eg, clinicians or payers) in the design of the study.

Not Applicable.

**Results**

**22. Study parameters**

Report all analytic inputs (eg, values, ranges, references) including uncertainty or distributional assumptions.

See Results, Tables 2 and 3, and Tables S1–S9.

**23. Summary of main results**

Report the mean values for the main categories of costs and outcomes of interest and summarize them in the most appropriate overall measure.

See Results and Tables 2, 3, 4, 5 and 6.

**24. Effect of uncertainty**

Describe how uncertainty about analytic judgments, inputs, or projections affects findings. Report the effect of choice of discount rate and time horizon, if applicable.

Population weights calculated by the The Finnish Institute for Health and Welfare were applied to the analyses to account for the sampling and non-participation of the survey participants. Appropriate statistical methods were applied to the analyses, see Methods.

**25. Effect of engagement with patients and others affected by the study**

Report on any difference patient/service recipient, general public, community, or stakeholder involvement made to the approach or findings of the study.

Not Applicable.

**Discussion**

**26. Study findings, limitations, generalizability, and current knowledge**

Report key findings, limitations, ethical, or equity considerations not captured and how these could impact patients, policy, or practice.

See Methods: Informed consent and ethical approval, and Discussion.

**Other Relevant Information**

**27. Source of funding**

Describe how the study was funded and any role of the funder in the identification, design, conduct, and reporting of the analysis.

This study was supported by Tampereen kaupungin tiederahasto, Tampere, Finland; Sokeain Ystävät - De Blindas Vänner sr., Helsinki, Finland; The Finnish Medical Society Duodecim, Helsinki, Finland; and Silmäsäätiö sr., Helsinki, Finland. The funders had no role in the design and conduct of the study; collection, management, analysis, and interpretation of the data; preparation, review, or approval of the manuscript; and decision to submit the manuscript for publication.

**28. Conflicts of interest**

Report authors’ conflicts of interest according to journal or International Committee of Medical Journal Editors requirements.

Authors declare no conflicts of interest.
